# Discovery and validation of a robust 11-gene prognostic signature via integrative multi-omics profiling in VD-CAG-treated non-M3 AML

**DOI:** 10.3389/fonc.2026.1801526

**Published:** 2026-05-19

**Authors:** Jirui Tang, Kun Yang, Ying Zhang, Yuqian Tang, Yankun Yang, Qin Li, Xuemei Wang, Yuping Gong

**Affiliations:** 1 Department of Hematology, West China Hospital, Sichuan University, Chengdu, China; 2 Department of Hematology, The Affiliated Hospital of Southwest Medical University, Luzhou, China

**Keywords:** acute myeloid leukemia, overall survival, prognostic signature, therapeutic response, VD-CAG

## Abstract

**Objectives:**

While the venetoclax-based VD-CAG regimen has shown promising efficacy in acute myeloid leukemia (AML), its underlying molecular response mechanisms remain poorly understood, and robust prognostic biomarkers for non-M3 AML treated with this regimen are lacking. This study aimed to elucidate the molecular alterations associated with VD-CAG treatment response, and to identify and validate robust prognostic biomarkers to improve risk stratification for non-M3 AML patients.

**Methods:**

This study enrolled 15 non-M3 AML patients receiving VD-CAG induction therapy, and conducted integrative multi-omics profiling of their bone marrow samples: RNA sequencing was performed on 10 pairs of pre- and post-treatment samples, while data-independent acquisition (DIA) proteomics was applied to another 5 paired samples. Weighted gene co-expression network analysis (WGCNA) was used to identify gene modules correlated with composite complete remission. A prognostic signature was further derived using Least Absolute Shrinkage and Selection Operator (LASSO) regression, with the TCGA-LAML cohort as the discovery set and the independent Beat AML cohort as the external validation set.

**Results:**

Transcriptomic analysis revealed that differentially expressed genes after VD-CAG treatment were mainly enriched in MAPK signaling and cell adhesion pathways. WGCNA identified five gene modules significantly correlated with treatment remission. We finally constructed a robust 11-gene prognostic signature (*TLN1, ARL15, PDZD2, ACER2, IGF2BP3, TMEM200A, DOCK1, SYTL4, CPNE8, FNDC3B*, and *MANBA*), which effectively stratified patients' overall survival. This signature showed strong predictive performance in both the discovery cohort (TCGA: HR=3.60, *p*=7.6e-10; 3-year AUC=0.79) and the independent validation cohort (Beat AML: HR=1.89, *p*=1.3e-3; 3-year AUC=0.83). Proteomic analysis identified 447 differentially expressed proteins between pre- and post-treatment samples, among which FNDC3B, a key protein in the prognostic signature, was significantly downregulated after VD-CAG intervention.

**Conclusions:**

This study reveals the molecular alterations associated with VD-CAG treatment in non-M3 AML, and provides a validated 11-gene prognostic signature. These findings enhance our understanding of the response mechanisms of the VD-CAG regimen, and offer a tool to improve prognostic stratification and clinical decision-making for non-M3 AML patients.

## 1 Introduction

Acute myeloid leukemia (AML) remains the most common acute leukemia in adults, characterized by the uncontrolled proliferation of immature myeloid blasts in the bone marrow and peripheral blood, leading to bone marrow failure and life-threatening complications (1). In contrast to acute promyelocytic leukemia (APL or M3), non-M3 AML represents the majority of cases and presents unique treatment challenges, such as higher relapse risks and the need for intensive chemotherapy (2). Despite advances in therapeutic strategies, the prognosis for non-M3 AML patients is heterogeneous, with 5-year overall survival rates hovering below 50% for most adults (3). Traditional induction therapy with cytarabine plus an anthracycline (7 + 3 regimen) achieves complete remission (CR) in approximately 60-70% of newly diagnosed AML (ND-AML) patients under 60 years (3, 4), but relapse rates exceed 30-40%, particularly in those with adverse-risk cytogenetics or molecular abnormalities (5, 6).

The integration of venetoclax, a selective BCL-2 inhibitor, into frontline AML therapy has revolutionized treatment paradigms, particularly for older or unfit patients when combined with hypomethylating agents or low-dose cytarabine (7, 8). Building upon this success, recent studies have explored venetoclax-based combinations with modified intensive chemotherapy regimens, demonstrating promising efficacy with composite complete remission rates exceeding 90% in newly diagnosed patients (9–11).

We recently reported that the combination of venetoclax with decitabine, cytarabine, aclarubicin, and granulocyte colony-stimulating factor (VD-CAG) achieved an impressive composite complete remission rate of 86.9% in young, fit adults with newly diagnosed non-M3 AML, with 79% of responders achieving measurable residual disease negativity (12, 13). This regimen demonstrated favorable safety profiles with no treatment-related deaths, suggesting an optimal balance between efficacy and tolerability (12, 13). However, the molecular mechanisms underlying treatment response and the identification of patients most likely to benefit from VD-CAG induction remain incompletely understood. Moreover, robust prognostic biomarkers capable of predicting long-term survival outcomes in this treatment context are urgently needed to guide clinical decision-making and personalized therapeutic strategies.

In this study, we conducted integrative multi-omics profiling of bone marrow aspirate samples from 15 non-M3 AML patients before and after VD-CAG induction to dissect the molecular landscape of treatment response. Using weighted gene co-expression network analysis (WGCNA), we identified gene modules significantly associated with composite complete remission status. Through least absolute shrinkage and selection operator (LASSO) regression modeling in the TCGA-LAML (14) discovery cohort, with validation in an independent Beat AML dataset (15, 16), we systematically evaluated the prognostic value of response-related gene signatures. Finally, Data-Independent Acquisition Proteomics confirmed differential expression of key signature proteins in treatment responders.

## 2 Materials and methods

### 2.1 Patients

This study utilized a subset of patients from a previously reported multicenter, retrospective cohort of adults aged 18–60 years with newly diagnosed non-M3 acute myeloid leukemia (AML), including *de novo*, secondary, and therapy-related AML, as defined by WHO criteria. Clinical data were collected from the electronic medical records of West China Hospital and other participating centers during the study period from March 1, 2022, to December 31, 2024. Patients with acute promyelocytic leukemia or those unfit for intensive chemotherapy were excluded. Detailed eligibility criteria and ethical approvals are described as we previously reported (12). For the current analysis, we focused on patients with available bone marrow aspirate samples for transcriptomic and proteomic profiling, ensuring representation across response groups. This research obtained ethical approval from the Ethics Committee at West China Hospital, Sichuan University (Approval ID: 2024-1182) and was carried out in compliance with Good Clinical Practice standards and the principles outlined in the Declaration of Helsinki. Written informed consent for participation in the research was obtained from all patients.

### 2.2 Treatment

All patients received induction therapy with the venetoclax plus decitabine, cytarabine, aclarubicin, and granulocyte colony-stimulating factor (VD-CAG) regimen, followed by risk-stratified consolidation therapy (e.g., high-dose cytarabine with venetoclax or allogeneic hematopoietic stem cell transplantation) based on Chinese AML guidelines. Full treatment protocols, including dosing and supportive care, are detailed in our prior publication (12). In brief, All patients received induction therapy with the VD-CAG regimen, which consisted of: oral venetoclax (100 mg on day 1, 200 mg on day 2, and 400 mg on days 3–9); intravenous decitabine (20 mg/m² on days 1–5); intravenous aclarubicin (10 mg/m² on days 3–6); subcutaneous cytarabine (10 mg/m² every 12 hours on days 3–9); and subcutaneous G-CSF (300 μg/day from day 1 to day 9, adjusted based on white blood cell count). Specifically, G-CSF was withheld if the WBC count exceeded 10 × 10^9^/L at baseline or 20 × 10^9^/L during the cycle.

Comprehensive supportive care was provided to ensure patient safety and treatment replication. Tumor lysis syndrome (TLS) was prevented through aggressive hydration and the administration of allopurinol or rasburicase based on baseline uric acid levels. Antimicrobial prophylaxis followed institutional standards, typically including a fluoroquinolone, an antiviral (e.g., acyclovir), and an antifungal agent (e.g., posaconazole or voriconazole). To manage drug-drug interactions, the venetoclax dose was adjusted when co-administered with CYP3A4 inhibitors: reduced to 70–100 mg (approximately 75-80% reduction) for strong inhibitors (e.g., posaconazole) and by 50% for moderate inhibitors. Transfusion thresholds were strictly maintained, with packed red blood cells administered for hemoglobin <70 g/L and platelet transfusions for counts <10 × 10^9^/L (or <20 × 10^9^/L in the presence of fever or bleeding).

Clinical response was assessed after one cycle of induction therapy according to the modified International Working Group (IWG) response criteria for AML. The primary clinical trait used for correlation analysis, composite complete remission (CRc), was defined as the sum of complete remission (CR) and complete remission with incomplete hematologic recovery (CRi). CR required a bone marrow regenerative response with <5% blasts, absence of blasts with Auer rods, absence of extramedullary disease, absolute neutrophil count >1.0 × 10^9^/L, and platelet count >100 × 10^9^/L. CRi was defined as meeting all CR criteria except for residual neutropenia (<1.0 × 10^9^/L) or thrombocytopenia (<100 × 10^9^/L). Measurable residual disease (MRD) was evaluated via 10-color flow cytometry on bone marrow aspirates, with a sensitivity of 0.01%. MRD negativity (MRD-) was defined as <0.01% detectable leukemia-associated immunophenotypes (LAIPs) or different-from-normal (DfN) cell populations. Patients achieving CR/CRi received risk-stratified consolidation therapy comprising 4 cycles of high-dose cytarabine (2 g/m² every 12h, days 1–3) combined with venetoclax (400 mg, days 1–7) or allogeneic hematopoietic stem cell transplantation (allo-HSCT) according to MRD status and donor availability.

### 2.3 RNA sequencing and data processing

RNA Sequencing was conducted by Novogene (Beijing, China), using paired bone marrow aspirate samples from 10 non-M3 AML patients before and after VD-CAG treatment. The detailed clinicopathological, cytogenetic, and molecular profiles of these patients, including their ELN 2022 risk stratification and specific mutation patterns, are comprehensively summarized in **Table 1**. Total RNA was extracted from bone marrow aspirate samples using standard protocols and assessed for integrity and quantity with the Agilent 2100 Bioanalyzer. mRNA was enriched either by poly(A) selection using Oligo(dT) magnetic beads or by ribosomal RNA depletion. Fragmented mRNA was reverse transcribed to cDNA, followed by second-strand synthesis, end repair, A-tailing, adaptor ligation, and PCR amplification to construct sequencing libraries. Library quality was evaluated by Qubit fluorometry and Agilent Bioanalyzer, and libraries were pooled according to effective concentration and target data yield. Sequencing was performed on the Illumina platform using the sequencing-by-synthesis method. Raw data were processed for quality control, and gene expression levels were quantified as fragments per kilobase of transcript per million mapped reads (FPKM).

**Table 1:** The clinicopathological parameters of 15 AML patients included.

Sample	FAB	VD-CAG response	Karyotype (ISCN)	Cytogenetics / molecular alterations	ELN 2022 risk	Analysis method
A	M2	CRc	46,XY[20]	*Biallelic CEBPA* mutations	Favorable	RNA-seq
B	M5	CRc	46,XX[20]	*NPM1*, *DNMT3A*, *FLT3*-TKD, *NRAS* mutations	Favorable	RNA-seq
C	M4	CRc	46,XY[20]	*KMT2A::ELL* fusion; *EP300* mutation	Adverse	RNA-seq
D	M4	CRc	46,XY,add(11)(p15),del(20)(q11.2)[20]	*EVI1* overexpression; *FLT3*-ITD; *NRAS* mutation	Adverse	RNA-seq
E	M2	CRc	46,XX[20]	*Biallelic CEBPA* mutations; *NRAS* mutation	Favorable	RNA-seq
F	M5	CRc	47,XX,t(11;19)(q23;p13.1),+mar[12]/46,XX[8]	*STAG2* mutation	Adverse	RNA-seq
G	M2	CRc	46,XX,add(9)(q13)[18]/46,XX[2]	*Biallelic CEBPA*, *EP300*, *GATA2* mutations	Favorable	RNA-seq
H	M5	CRc	46,XX,t(5;6)(q35;q25)[20]	*IDH2* mutation	Intermediate	RNA-seq
I	M4	CRc	45,XX,−10,der(11)t(10;11)(q21;q21)[19]/46,XX[1]	*TP53* (Class II), *NRAS* mutations	Adverse	RNA-seq
J	M4	CRc	46,XX[20]	*CEBPA*, *DHX15*, *GATA2* mutations	Adverse	RNA-seq
P1	M4	CRc	46,XX[20]	*NUP98::NSD1* fusion; *FLT3*, *RAS* pathway, *WT1*, *MYC* mut.	Intermediate	DIA Proteomics
P2	M4	CRc	46,XY,inv(16)(p13q22)[9]/47,idem,+22[3]	*FLT3*-ITD; *FLT3*, *KIT* alterations	Adverse	DIA Proteomics
P3	M4	CRc	47,XY,−7,inv(16)(p13q22),+21,+r	*CBFB::MYH11* fusion; *NRAS*, *WT1* mutations	Adverse	DIA Proteomics
P4	M2	CRc	Complex karyotype (including del(5q), −7, +8)	*TP53* mutation	Adverse	DIA Proteomics
P5	M2	CRc	46,XY[20]	*CEBPA* (non-bZIP), *TET2* mutations; *WT1* high expression	Intermediate	DIA Proteomics

All samples were collected via bone marrow aspirate (BMA). FAB, French-American-British classification; VD-CAG, Venetoclax, Decitabine, Cytarabine, Aclarubicin, and G-CSF; CRc, Composite complete remission (CR + CRi); ISCN, International System for Human Cytogenomic Nomenclature; ELN, European LeukemiaNet (2022 criteria); RNA-seq, Whole transcriptome sequencing; DIA Proteomics, Data-independent acquisition mass spectrometry-based proteomics; *Biallelic CEBPA* mutations refer to double mutations (typically involving bZIP domain as per ELN 2022).

Bioinformatics analyses were performed using R software (version 4.2.0) and associated packages. Sample quality and reproducibility were assessed via Pearson correlation analysis (R package “corrplot”) to generate heatmaps, with R² > 0.8 required for biological replicates. Principal component analysis (PCA) was conducted on FPKM values using the “prcomp” function to evaluate inter- and intra-group variability. To ensure statistical rigor, differential expression analysis was performed using raw read counts as input for DESeq2 (version 1.34.0). A subject-blocked design matrix (Patient + Treatment) was utilized to explicitly model the paired pre/post-induction design, thereby controlling for inter-patient heterogeneity. Differentially expressed genes (DEGs) were identified based on |log2FoldChange| > 1 and adjusted *p*-value (padj) < 0.05. Volcano plots were generated using the “ggplot2” package to visualize DEGs. Hierarchical clustering of DEGs was performed with the “pheatmap” package, applying Z-score normalization to log_2_-transformed FPKM values for heatmap visualization, including annotations for chromosome location, gene length, and biotype. Functional enrichment analyses included Gene Ontology (GO) using the “clusterProfiler” package (version 4.2.2), with terms categorized into biological process, cellular component, and molecular function; KEGG pathway enrichment via “clusterProfiler”; and Reactome pathway analysis using the “ReactomePA” package (version 1.38.0). To provide a comprehensive overview of the global molecular shifts induced by VD-CAG therapy, these enrichment analyses were performed on the total set of DEGs (including both upregulated and downregulated genes simultaneously). Enrichment significance was determined at padj < 0.05, and results were visualized as scatterplots or bar plots for the top enriched terms/pathways.

### 2.4 Proteomic analysis

Proteomic profiling was performed by Novogene (Beijing, China), using data-independent acquisition (DIA) proteomics on paired bone marrow aspirate samples from 5 non-M3 AML patients before and after VD-CAG treatment (see **Table 1** for sample details). Protein extraction, quantification, enzymatic digestion with trypsin (allowing up to 2 missed cleavage sites), desalting, and fractionation were conducted following standard protocols to ensure data quality at each step. Peptides were separated using nano-LC on a Vanquish Neo system and analyzed on an Orbitrap Astral high-resolution mass spectrometer, which features a quadrupole for precursor ion selection, an Orbitrap analyzer for high-dynamic-range HRAM spectra, and an Astral analyzer for high-sensitivity, up to 200 Hz HRAM spectral acquisition.

Raw MS data (.raw files) were processed using DIA-NN 1.8.1 software for database searching against the UniProt human database (homo_sapiens_uniprot_2025_06_11_Swissprot.fasta), with fixed modification of carbamidomethylation (C) and variable modification of N-terminal methionine excision. Protein identification required a global precursor q-value < 0.01 and protein group q-value < 0.01, resulting in 48,272 peptides and 6,830 identified and quantified proteins across all samples.

Data quality control included assessment of peptide length distribution, protein coverage, and sample reproducibility, with high intra-group Pearson correlation (R² > 0.8). Principal component analysis (PCA) was conducted on the quantified protein expression matrix to evaluate group separation. Differential expression analysis of proteins (DEPs) was conducted using fold change > 2.0 (upregulated > 2.0-fold or downregulated < 0.5-fold) and *p*-value < 0.05 as criteria. Functional annotation of all identified proteins was performed using KEGG, InterPro (for protein domains), and subcellular localization analysis. Consistent with the transcriptomic analysis, functional enrichment of DEPs was performed on the integrated set of all differentially expressed proteins.

### 2.5 Weighted gene co-expression network analysis

UCSC Xene browser (17) and Vizome (https://www.vizome.org/) were used to get expression and survival data of TCGA-LAML (n=133) and Beat (n=200) respectively. WGCNA was performed using the R package “WGCNA” (version 1.72-1) to identify gene modules associated with composite complete response (CRc). Genes were first filtered by median absolute deviation (MAD), retaining the top 50% most variable genes. Outlier genes and samples were removed using the goodSamplesGenes function. A weighted adjacency matrix was constructed using Pearson correlation coefficients raised to a soft-thresholding power (β), selected based on scale-free topology criteria (R² > 0.85). For this analysis, β = 22 was chosen. The adjacency matrix was transformed into a topological overlap matrix (TOM), and hierarchical clustering was performed to group genes into modules with a minimum module size of 30. Modules with eigengene dissimilarity less than 0.25 were merged. Module eigengenes were correlated with clinical traits, including treatment status and CRc, to identify modules of interest. The grey module contained genes not assigned to any specific module.

### 2.6 LASSO regression and prognostic model construction

Prognostic signatures were constructed using LASSO Cox regression implemented in the R package “glmnet” (version 4.1-4). For each WGCNA module of interest, genes were used as candidate predictors for overall survival (OS) in the TCGA-LAML cohort (non-M3 AML cases). Samples with missing survival data or clinical information were excluded from the respective analyses. The penalty parameter (λ) was determined via 10-fold cross-validation, which was repeated 100 times to ensure the stability of gene selection, using a fixed random seed (2024) for reproducibility. The resulting gene signatures were used to calculate risk scores for patient stratification. The proportional hazards (PH) assumption for the Cox models was evaluated and confirmed using Schoenfeld residual tests (cox.zph). Time-dependent receiver operating characteristic (ROC) analysis was performed using the “timeROC” package (version 0.4) to evaluate predictive accuracy at 1-, 3-, and 5-year time points. Survival analysis and Kaplan-Meier curves were generated using the “survival” and “survminer” packages (version 0.4.9). Univariate and multivariate Cox proportional hazards regression analyses were conducted to assess the independent prognostic value of the signatures, adjusting for age, sex, cytogenetic risk, and other clinicopathological variables.

### 2.7 Incremental value analyses (C-index change, NRI/IDI, and decision curve analysis)

Incremental prognostic performance of the 11-gene signature was assessed by comparing a baseline clinicogenetic Cox model (Age + Cytogenetics) with an expanded model additionally including the 11-gene risk score. Cox proportional hazards models were fitted using the R package survival (v3.3.1) in R v4.2.1. Model discrimination was evaluated using time-dependent C-index. The statistical significance of the change in C-index (ΔC-index, mean ± SD) between the baseline and expanded models was evaluated at 1, 2, and 3 years.

Reclassification performance at the 3-year horizon was quantified by IDI using survIDINRI (v1.1.2) with 300 resamples (seed = 2024) and by NRI using nricens (v1.6) with 200 resamples (seed = 2024). Visualizations were generated with ggplot2 (v3.4.4).

Clinical utility was evaluated using decision curve analysis (DCA) at 1-year and 3-year horizons based on fitted Cox models, implemented with a customized stdca.R script, reporting net benefit across threshold probabilities.

### 2.8 Statistical analysis

Statistical analyses were conducted in R software (version 4.2.0) or GraphPad Prism 10.1.2. Comparisons of continuous variables employed Student’s t-test or the Wilcoxon rank-sum test, depending on data distribution. For categorical variables, the chi-square test or Fisher’s exact test was utilized. Survival differences were assessed via the log-rank test. Cox proportional hazards regression was used to estimate hazard ratios (HR) along with their 95% confidence intervals (CI). A *p*-value threshold of less than 0.05 indicated statistical significance. The Benjamini-Hochberg procedure was implemented for multiple comparison adjustments when necessary.

## 3 Results

### 3.1 Study design and patient characteristics

**Figure 1:**
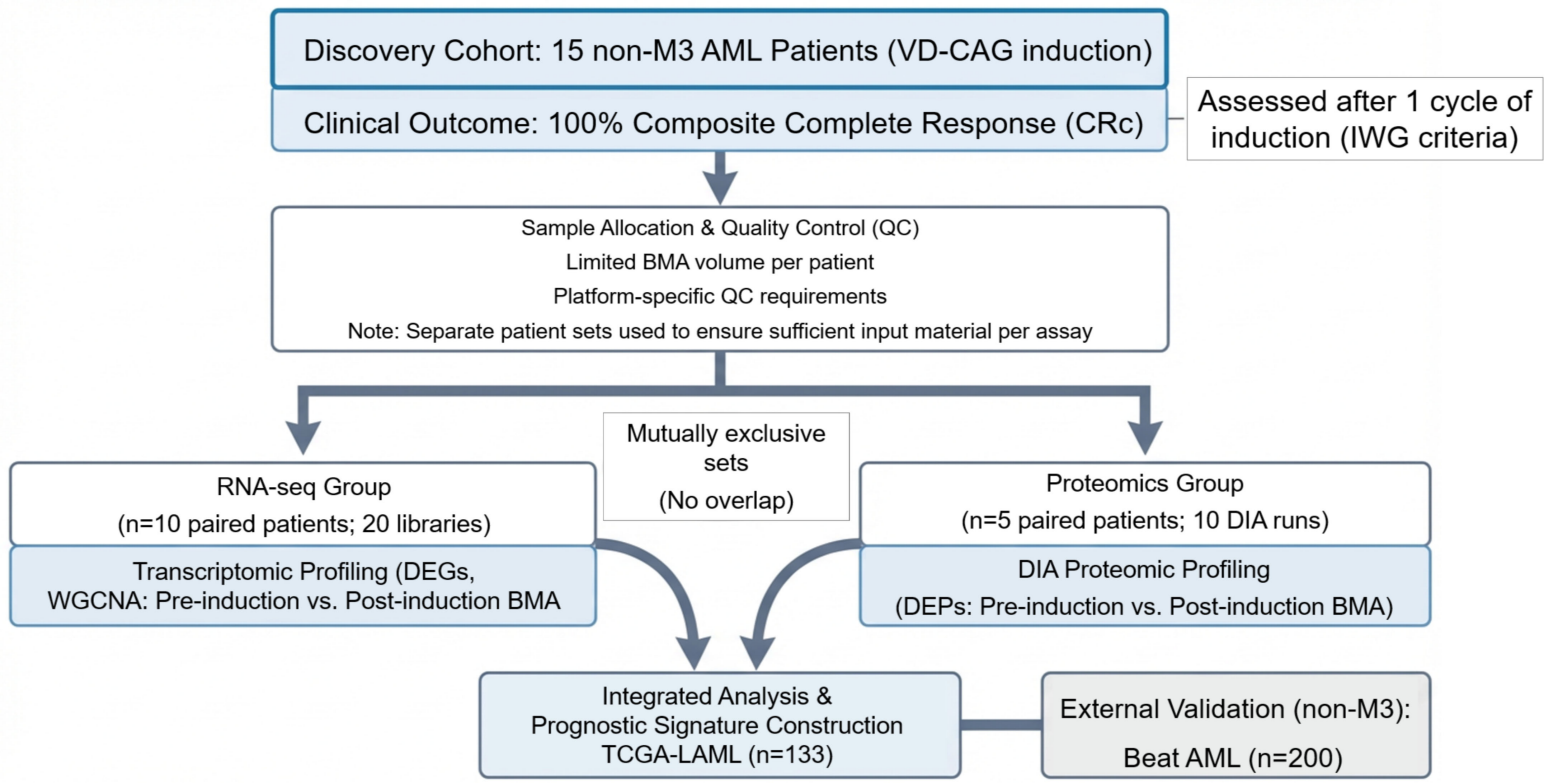
Schematic overview of the study design. A discovery cohort of 15 non-M3 AML patients received VD-CAG induction therapy (venetoclax plus decitabine, cytarabine, aclarubicin, and granulocyte colony-stimulating factor), and all achieved composite complete response (CRc) after one induction cycle (IWG criteria). Due to limited bone marrow aspirate (BMA) volume and platform-specific quality control requirements, omics assays were performed in two mutually exclusive paired-sample sets: RNA-seq (n=10 paired patients; 20 libraries) and DIA proteomics (n=5 paired patients; 10 DIA runs), comparing pre-induction versus post-induction BMA. Multi-omics results were integrated for prognostic signature construction in the non-M3 TCGA-LAML cohort (n=133) and externally validated in the non-M3 Beat AML cohort (n=200).

To elucidate the molecular changes associated with successful induction therapy and subsequent survival outcomes in non-M3 AML patients, we performed integrative transcriptomic and proteomic profiling of bone marrow aspirate (BMA) samples collected pre-induction and post-induction (**Figure 1**). The clinicopathological information of the 15 patients was included in **Table 1**. All 15 patients achieved composite complete response (CRc) after 1 cycle of VD-CAG induction, assessed according to IWG criteria (**Figure 1**). Due to limited BMA volume per patient and platform-specific quality control (QC) requirements, the cohort was divided into two mutually exclusive paired-sample sets: RNA-seq (n=10 paired patients; 20 libraries) and DIA proteomics (n=5 paired patients; 10 DIA runs) (**Figure 1**). Using WGCNA and LASSO regression, we aimed to identify candidate gene sets and construct a potential prognostic gene signature that could be further validated for its ability to stratify patients by overall survival (OS).

### 3.2 RNA-seq and pathway enrichment

**Figure 2:**
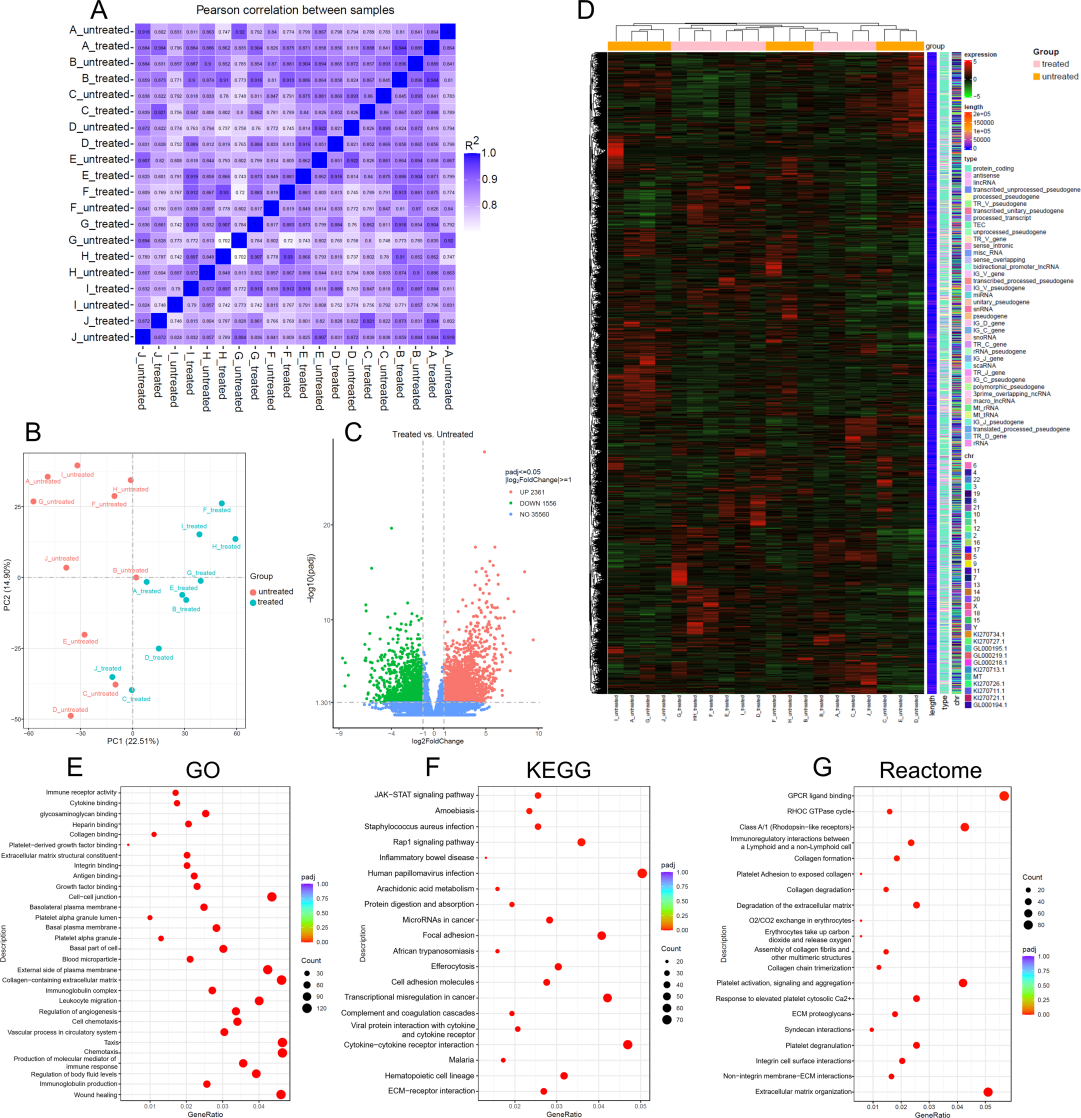
Transcriptomic profiling and quality assessment of bone marrow aspirate samples before and after VD-CAG induction **(A)** Pearson correlation heatmap of gene expression profiles across all samples. **(B)** principal component analysis (PCA) plot based on FPKM values, showing clear separation between treated and untreated groups. **(C)** volcano plot illustrating the distribution of differentially expressed genes (DEGs) between treated and untreated samples. Red dots represent upregulated genes, green dots represent downregulated genes, and blue dots indicate non-significant genes. **(D)** hierarchical clustering heatmap of DEGs, normalized by Z-score across samples. Genes with similar expression patterns are grouped together, and sample clustering reflects group assignment. Additional annotations include chromosome location, gene length, and gene biotype. **(E)** gene ontology (GO) enrichment analysis of DEGs, displaying the top 30 significantly enriched GO terms. Dot size indicates the number of genes annotated to each term, and color represents enrichment significance. **(F)** KEGG pathway enrichment analysis of DEGs, showing the top 20 significantly enriched pathways. Dot size and color indicate the number of genes and enrichment significance, respectively. **(G)** reactome pathway enrichment analysis of DEGs, presenting the top 20 significantly enriched reactome pathways. Bar height and color reflect enrichment significance, with numbers indicating the count of DEGs in each pathway.

To assess the quality and consistency of transcriptomic data, we first evaluated the correlation of gene expression profiles among biological replicates. Pearson correlation analysis revealed high intra-group similarity (R² > 0.8 for all samples), indicating robust reproducibility and reliable sample selection (**Figure 2A**). Principal component analysis (PCA) further confirmed distinct separation between pre-induction and post-induction groups, with replicates clustering tightly within each group (**Figure 2B**).

Differential expression analysis identified a substantial number of genes significantly upregulated or downregulated following VD-CAG induction (**Figure 2C**, **Supplementary Table 1**). Hierarchical clustering of DEGs, normalized by Z-score, demonstrated clear group-specific expression patterns and further validated the consistency of biological replicates (**Figure 2D**).

Functional enrichment analyses were performed on the integrated set of all DEGs to elucidate the biological significance of the observed transcriptomic changes. GO enrichment analysis highlighted terms related to immune receptor activity, cell adhesion, and apoptotic signaling among the top enriched categories (**Figure 2E**). KEGG pathway analysis revealed significant enrichment in pathways such as MAPK signaling, cytokine-cytokine receptor interaction, and cell adhesion molecules (**Figure 2F**). Reactome pathway analysis identified GPCR signaling, extracellular matrix organization, and integrin cell surface interactions as prominent pathways affected by treatment (**Figure 2G**).

### 3.3 Key gene modules related to VD-CAG responses

**Figure 3:**
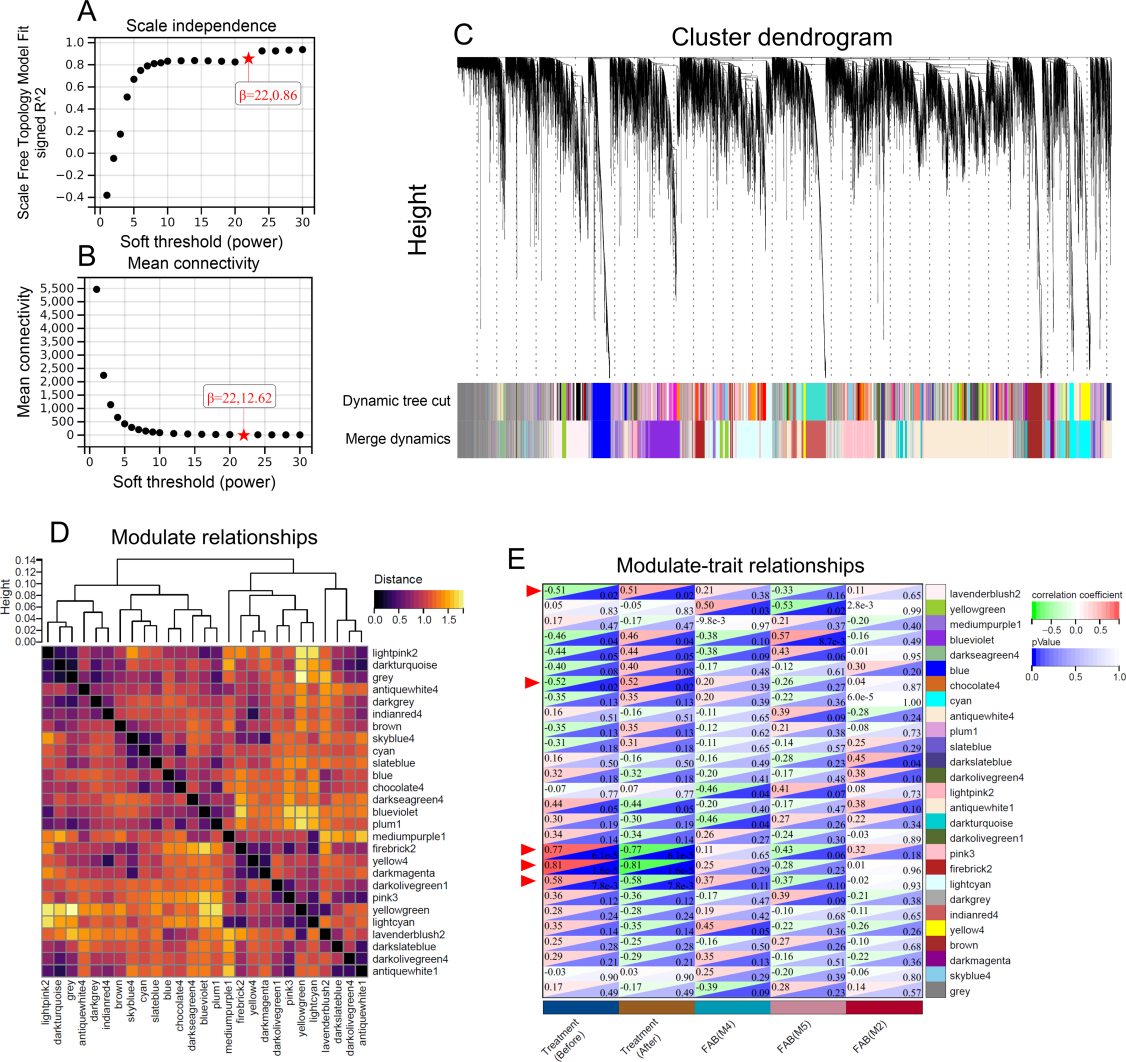
WGCNA identifies key gene modules associated with CRc in AML. **(A)** analysis of scale-free topology fit index (y-axis) as a function of the soft-thresholding power (x-axis). The optimal power value (β = 22) was selected to achieve a scale-free R² > 0.85. **(B)** mean connectivity as a function of the soft-thresholding power, showing the decrease in connectivity with increasing power. **(C)** hierarchical clustering dendrogram of genes based on topological overlap, with modules identified by dynamic tree cut and subsequent merging of similar modules. Each color represents a distinct gene module. **(D)** module eigengene dendrogram and heatmap showing relationships and distances among the identified modules. **(E)** heatmap of module-trait relationships, displaying the correlation coefficients and *p*-values between module eigengenes and clinical traits, with treatment status (pre-induction vs. post-induction) as the primary trait. Red arrowheads indicate modules most strongly associated with treatment status.

To capture the dynamic molecular shifts associated with successful induction, an exploratory WGCNA was performed using treatment status (pre-induction vs. post-induction BM) as the primary clinical trait in our cohort of 100% CRc responders. After filtering out 50% of genes with the lowest MAD, outlier genes and samples were removed using the goodSamplesGenes function. A scale-free network topology was achieved by selecting a soft-thresholding power of β = 22, which resulted in a scale-free topology fit index (R²) of 0.86 (**Figure 3A**) and acceptable mean connectivity (**Figure 3B**). Hierarchical clustering based on the TOM identified 27 candidate gene modules, with modules of similar expression profiles merged at a dissimilarity threshold of 0.25 (**Figures 3C, D**). The grey module represented genes not assigned to any specific module. Module-trait relationship analysis revealed five modules (firebrick2, pink3, Lightcyan, chocolate4, and lavenderblush2) significantly correlated with treatment status (pre-induction vs. post-induction) (**Figure 3E**, Pearson’s r>0.5, red arrowheads, **Supplementary Table 2**). Given the limited size of the discovery cohort, these modules were treated as preliminary candidate sets requiring rigorous external validation to confirm their biological stability and prognostic relevance.

### 3.4 Prognostic value of the VD-CAG response-related gene modules in non-M3 AML

To further investigate the prognostic relevance of gene modules identified by WGCNA, we focused on the top 5 modules. Using the TCGA-LAML cohort (excluding M3 cases) as the discovery dataset, we applied LASSO regression for model constructions and further validate each prognostic model in Beat dataset.

Based on the Firebrick2 module genes, we constructed a 7-gene prognostic signature (*POLG, TMED4, ZNF444, PICK1, COX10, GEMIN7, WDR83*) (**Supplementary Figures 1A–C**). The model signature is Risk score=0.32581996464363*POLG-0.449661099037936*TMED4 + 0.0566379144509434*ZNF444 + 0.117448150269237*PICK1-0.163202457986264*COX10 + 0.22654232707814*GEMIN7 + 0.096623486828942*WDR83. Patients were stratified into high- and low-risk groups based on the median calculated risk scores. The 7-gene signature can predict OS in TCGA-LAML (non-M3), with high-risk patients exhibiting significantly poorer outcomes (HR = 2.48, 95% CI: 1.60–3.84, *p* = 2.7e-5; **Supplementary Figure 1D**), with AUCs of 0.72, 0.78, and 0.84 at 1, 3, and 5 years in time-dependent ROC analysis (**Supplementary Figure 1E**). However, validation in the Beat AML cohort (non-M3) showed the prognostic utility of the 7-gene signature is not ideal (HR = 1.48, 95% CI: 1.02–2.18, *p* = 0.05; **Supplementary Figure 1F**). The signature showed moderate AUCs of 0.61 and 0.71 at 1 and 3 years, respectively (**Supplementary Figure 1G**).

Using Pink3 module genes, we identified a 4-gene signature (ZADH2, DYM, TMEM106A, FAM120C) (**Supplementary Figures 2A–C**), for the following model: Risk score=-0.246721428572035*ZADH2-0.197618185641332*DYM-0.01229384207435*TMEM106A-0.0355394026273012*FAM120C. Kaplan-Meier analysis demonstrated that the 4-gene signature could significantly stratify OS in TCGA-LAML (non-M3), with high-risk patients exhibiting worse outcomes (HR = 1.76, 95% CI: 1.15–2.70, *p* = 7.9e-3; **Supplementary Figure 2D**). Time-dependent ROC analysis showed moderate predictive accuracy, with AUCs of 0.68, 0.65, and 0.64 at 1, 3, and 5 years, respectively (**Supplementary Figure 2E**). Validation in the Beat AML cohort (non-M3) showed that the signature remained prognostic in Beat AML (HR = 1.59, 95% CI: 1.08–2.35, *p* = 0.02; **Supplementary Figure 2F**), with AUCs of 0.63 and 0.75 at 1 and 3 years, respectively (**Supplementary Figure 2G**).

Using the Lightcyan module genes, LASSO regression identified a 13-gene signature (*LGALS1, CLEC11A, FPK1, TRIM27, EBP, MPP1, CHEB3, ETFB, DHRSX, PPAP4, TAP1, ATP6V1, IL17RE*) (**Supplementary Figures 3A–C**), with the model signature is: Risk score=0.000998358118049984*LGALS1-0.144694627876313*CLEC11A + 0.163553872288672*PFKL-0.235886401281219*TRIM27 + 0.0177880897471083*EBP + 0.0747088075877768*MPI + 0.133677503131668*CREB3 + 0.174117106842282*ETFB-0.374161851062922*DHRSX-0.0141895295238673*PAQR4 + 0.611124094355214*TFPT-0.0421662277260342*AP4M1 + 0.0259629898690867*IL17RE.

The 13-gene signature robustly stratified OS in TCGA-LAML (non-M3), with high-risk patients exhibiting significantly poorer outcomes (HR = 4.89, 95% CI: 3.06–7.81, *p* = 8.4e-13; **Supplementary Figure 3D**). Time-dependent ROC analysis showed strong predictive accuracy, with AUCs of 0.80, 0.87, and 0.94 at 1, 3, and 5 years, respectively (**Supplementary Figure 3E**). Despite this exceptional discovery performance, validation in the Beat AML cohort (non-M3) showed that the 13-gene signature lost prognostic utility (HR = 1.42, 95% CI: 0.97–2.09, *p* = 0.07; **Supplementary Figure 3F**), with AUCs of 0.57 and 0.69 at 1 and 3 years, respectively (**Supplementary Figure 3G**).

**Figure 4:**
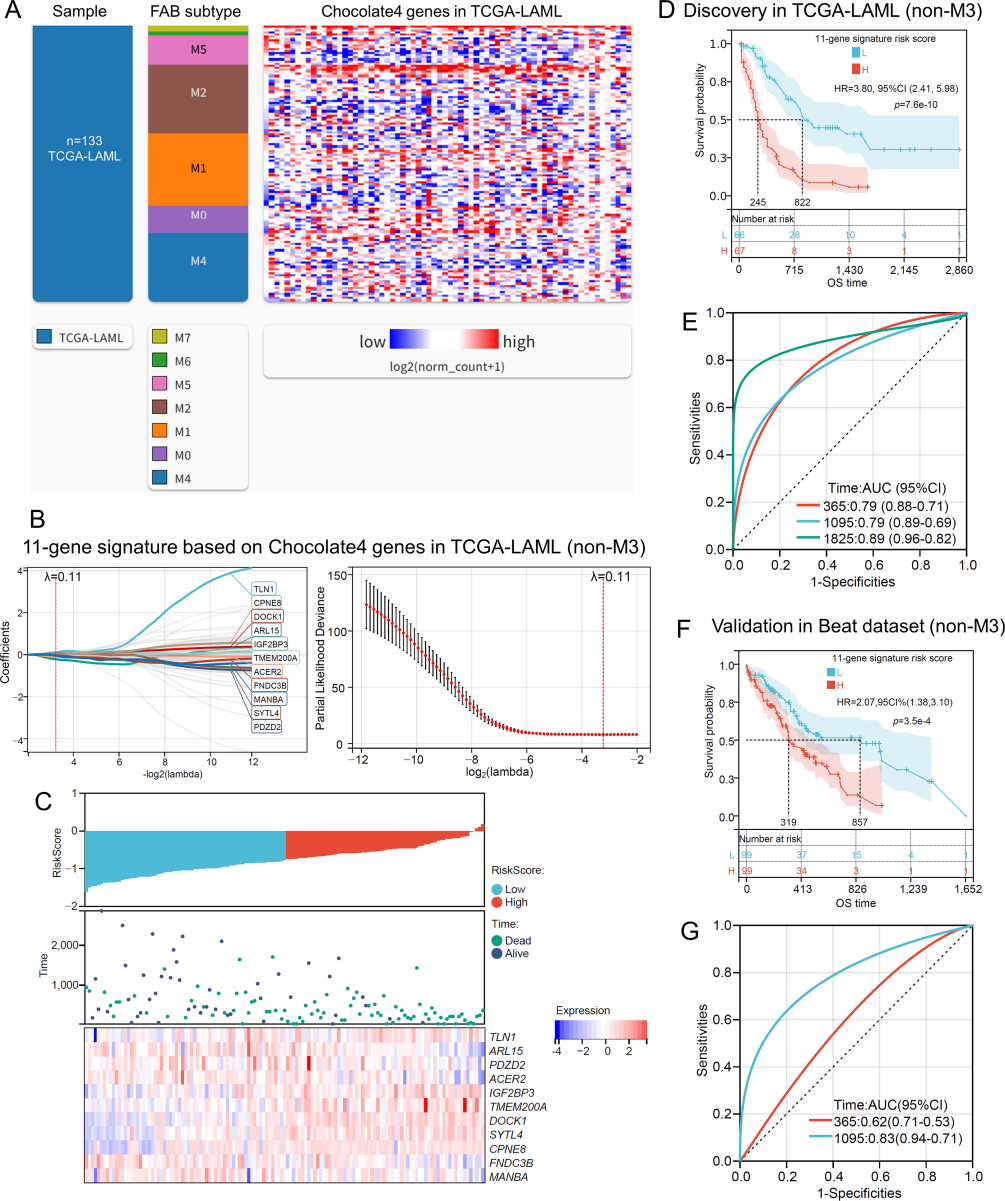
Construction and validation of a prognostic gene signature for overall survival in non-M3 AML using Chocolate4 module genes. **(A)** heatmap showing Chocolate4 module gene expression across non-M3 FAB subtypes in the TCGA-LAML cohort (n=133). **(B)** LASSO regression analysis in TCGA-LAML (non-M3) identifies an 11-gene prognostic signature from Chocolate4 module genes. Left: LASSO coefficient profiles; right: cross-validation plot for optimal lambda selection. **(C)** risk score distribution, survival status, and expression heatmap of the 11 signature genes in TCGA-LAML (non-M3). **(D)** Kaplan-Meier survival curves for OS in TCGA-LAML (non-M3) stratified by the median 11-gene signature risk score. **(E)** time-dependent ROC curves for the 11-gene signature in TCGA-LAML (non-M3), showing AUCs at 1, 3, and 5 years. **(F)** Kaplan-Meier survival curves for OS in the Beat AML validation cohort (non-M3) stratified by the median 11-gene signature risk score. **(G)** time-dependent ROC curves for the 11-gene signature in the Beat AML validation cohort (non-M3), showing AUCs at 1 and 3 years.

Using the Chocolate4 module genes, LASSO regression identified an 11-gene signature (*TLN1, ARL15, PDZD2, ACER2, IGF2BP3, TMEM200A, DOCK1, SYTL4, CPNE8, FNDC3B, MANBA*) (**Figures 4A–C**). The model signature is:

Risk score=0.118677912111381*TLN1-0.219426877035103*ARL15-0.0565754778775297*PDZD2-0.101386399090571*ACER2 + 0.0467769345584947*IGF2BP3 + 0.0361612261769685*TMEM200A + 0.0261889356819606*DOCK1 + 0.0470245523311882*SYTL4 + 0.0176078860197474*CPNE8-0.0301792804525126*FNDC3B-0.0377966889919586*MANBA.

In the TCGA-LAML discovery cohort, the 11-gene signature effectively stratified patients by OS, with the high-risk group showing significantly worse outcomes (HR = 3.60, 95% CI: 2.41–5.98, *p* = 7.6e-10; **Figure 4D**). The time-dependent ROC analysis demonstrated strong predictive performance, with AUCs of 0.79, 0.79, and 0.89 at 1, 3, and 5 years, respectively (**Figure 4E**).

Validation in the Beat AML cohort (non-M3) confirmed the prognostic value of the 11-gene signature, as high-risk patients again had significantly poorer survival (HR = 1.89, 95% CI: 1.27–2.80, *p* = 1.3e-3; **Figure 4F**). The model also showed moderate predictive accuracy in the validation set, with AUCs of 0.83 at 3 years (**Figure 4G**).

Using the Lavenderblush2 genes, LASSO regression identified a 2-gene signature (*TSTA3*, and *ST6GALNAC6*) (**Supplementary Figures 4A–C**). The model signature is:

Risk score=0.0141968977005126*TSTA3 + 0.118042334476841*ST6GALNAC6. In the TCGA-LAML discovery cohort, the 2-gene signature significantly stratified patients by overall survival, with the high-risk group showing poorer outcomes (HR = 1.98, 95% CI: 1.29–3.04, *p* = 1.5e-3; **Supplementary Figure 4D**). Time-dependent ROC analysis showed moderate predictive performance, with AUCs of 0.67, 0.67, and 0.87 at 1, 3, and 5 years, respectively (**Supplementary Figure 4E**). However, validation in the Beat AML cohort (non-M3) did not confirm the prognostic value of the 2-gene signature, as the difference in survival between high- and low-risk groups was not statistically significant (HR = 1.29, 95% CI: 0.87–1.90, p=0.20; **Supplementary Figure 4F**). The model also showed limited predictive accuracy in the validation set, with AUCs of 0.49 and 0.69 at 1 and 3 years, respectively (**Supplementary Figure 4G**).

### 3.5 Univariate and multivariate analysis of the prognostic signature of the 11-gene signature

To further evaluate the prognostic value of the 11-gene signature, we performed univariate and multivariate Cox regression analyses for OS in the TCGA-LAML and Beat cohorts respectively, incorporating key clinicopathological variables (**Tables 2**, **3**). Uni- and Multi-variate Cox regression analysis confirmed that both age (HR = 1.022, 95% CI: 1.005–1.039, *p* = 0.011) and the 11-gene risk score stratification (HR = 0.306, 95% CI: 0.193–0.487, *p* < 0.001) remained independent prognostic factors for OS after adjusting for other clinical parameters in TCGA-LAML (**Table 3**). The independent prognostic value in the 11-gene signature was confirmed in the Beat dataset (HR = 0.631, 95% CI: 0.415–0.959, *p* = 0.031) (**Table 3**).

**Table 2:** Univariate and multivariate analysis of OS in TCGA-LAML.

Characteristics	Total (n)	Univariate analysis		Multivariate analysis	
		Hazard ratio (95% CI)	*p* value	Hazard ratio (95% CI)	*p* value
**FAB** **classification**	133				
M2	33	Reference			
M4	33	1.066 (0.588 - 1.934)	0.832		
M6	2	2.841 (0.655 - 12.331)	0.163		
M1	35	1.067 (0.582 - 1.958)	0.833		
M0	13	0.770 (0.339 - 1.752)	0.534		
M5	14	1.166 (0.530 - 2.566)	0.703		
M7	3	2.716 (0.798 - 9.245)	0.110		
**Gender**	133				
Male	72	Reference			
Female	61	1.014 (0.665 - 1.545)	0.950		
**Age at initial pathologic diagnosis**	133	1.031 (1.015 - 1.047)	**< 0.001**	1.022 (1.005 - 1.039)	**0.011**
*Cytogenetics risk category	131				
Adverse	26	Reference			
Intermediate	88	0.808 (0.476 - 1.373)	0.431		
Favorable	17	0.423 (0.176 - 1.018)	0.055		
**Bone marrow blast %**	133	0.999 (0.992 - 1.005)	0.668		
**WBC count (10^9^/L)**	99	1.003 (0.996 - 1.010)	0.364		
**Race**	132				
white	123	Reference			
Black or African American	8	1.275 (0.465 - 3.492)	0.637		
Asian	1	2.461 (0.338 - 17.910)	0.374		
**11-gene risk score**	133				
High	67	Reference		Reference	
Low	66	0.263 (0.167 - 0.414)	**< 0.001**	0.306 (0.193 - 0.487)	**< 0.001**

*Cytogenetic risk category developed by the Cancer and Leukemia Group B (CALGB). Bold indicates p<0.05.

**Table 3:** Univariate and multivariate analysis of OS in Beat.

Characteristics	Total (n)	Univariate analysis		Multivariate analysis	
		Hazard ratio (95% CI)	*p* value	Hazard ratio (95% CI)	*p* value
**FAB classification**	200				
M4	46	Reference		Reference	
M5	52	2.230 (1.269 - 3.921)	**0.005**	1.765 (0.988 - 3.151)	0.055
M2	43	1.174 (0.624 - 2.210)	0.619	1.186 (0.611 - 2.303)	0.614
M1	35	1.957 (1.022 - 3.748)	**0.043**	2.280 (1.162 - 4.471)	**0.016**
M0	18	1.551 (0.722 - 3.334)	0.261	0.749 (0.324 - 1.735)	0.501
M7	3	7.372 (2.140 - 25.395)	**0.002**	8.037 (2.208 - 29.258)	**0.002**
M6	3	4.737 (1.382 - 16.239)	**0.013**	5.074 (1.333 - 19.314)	**0.017**
**Gender**	200				
Female	93	Reference		Reference	
Male	107	1.404 (0.949 - 2.077)	0.089	1.211 (0.798 - 1.838)	0.369
**Age category**	200				
older	76	Reference		Reference	
young	56	0.514 (0.309 - 0.855)	**0.010**	0.408 (0.235 - 0.708)	**0.001**
middle	49	0.424 (0.246 - 0.732)	**0.002**	0.523 (0.291 - 0.938)	**0.030**
oldest	19	2.042 (1.179 - 3.536)	**0.011**	1.754 (0.963 - 3.195)	0.066
*Cytogenetics risk category	200				
Adverse	68	Reference		Reference	
Intermediate	65	0.694 (0.453 - 1.065)	0.094	0.822 (0.501 - 1.346)	0.435
Favorable	67	0.339 (0.199 - 0.577)	**< 0.001**	0.514 (0.274 - 0.963)	**0.038**
**Bone marrow blast %**	139	1.002 (0.992 - 1.011)	0.701		
**WBC count (10^9^/L)**	178	1.002 (0.999 - 1.005)	0.126		
**Race**	200				
White	176	Reference			
Asian	7	0.938 (0.344 - 2.556)	0.900		
HispNative	16	0.835 (0.337 - 2.068)	0.697		
Black	1	0.000 (0.000 - Inf)	0.995		
**11-gene signature score**	200				
High	100	Reference		Reference	
Low	100	0.529 (0.357 - 0.785)	**0.002**	0.631 (0.415 - 0.959)	**0.031**

* by ELN (European Leukemia Net) 2017 risk classification.Bold indicates *p*<0.05.

### 3.6 Incremental prognostic value beyond clinicogenetic model (age + cytogenetics).

To assess whether the 11-gene signature provides incremental predictive value beyond established clinicogenetic factors, we compared a baseline Cox model including age and cytogenetic risk with an expanded model additionally incorporating the 11-gene risk score in the TCGA-LAML (non-M3) cohort and the Beat AML validation cohort (**Supplementary Figure 5**). In TCGA-LAML, adding the 11-gene risk score significantly improved model discrimination (ΔC-index = 0.075 ± 0.003, *p* < 0.001; **Supplementary Figure 5A**). A smaller yet significant improvement was also observed in Beat AML (ΔC-index = 0.016 ± 0.004, *p* = 0.034; **Supplementary Figure 5D**).

At the 3-year horizon, reclassification analysis showed positive NRI values in both cohorts (TCGA-LAML: NRI = 0.146; Beat AML: NRI = 0.216), although the confidence intervals crossed zero (**Supplementary Figure 5B**). Discrimination improvement quantified by IDI demonstrated a significant gain in TCGA-LAML (IDI = 0.110, 95% CI 0.027–0.214, *p* = 0.007), whereas the improvement was not statistically significant in Beat AML (IDI = 0.060, 95% CI −0.015–0.156, *p* = 0.156) (**Supplementary Figure 5E**).

Decision curve analysis further indicated that incorporating the 11-gene signature yielded higher net benefit than cytogenetic stratification alone across a range of clinically relevant threshold probabilities at both 1 and 3 years in the discovery and validation cohorts (**Supplementary Figure 5C, F**), supporting the clinical utility of the signature as an adjunct to standard clinicogenetic risk assessment.

### 3.7 Proteomic data and pathway enrichment

**Figure 5:**
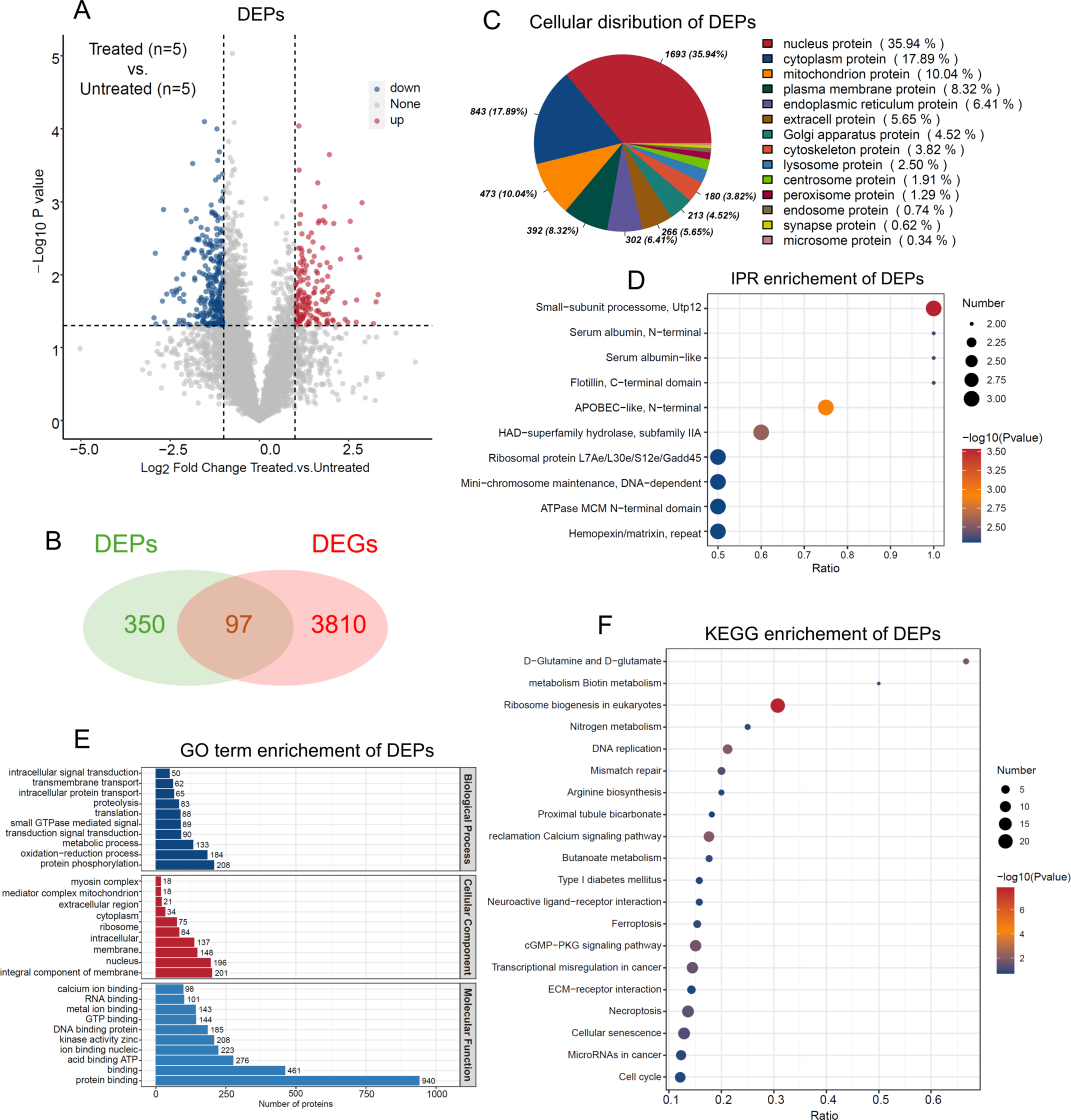
Proteomic profiling and pathway enrichment analysis of VD-CAG-treated non-M3 AML patients. **(A)** Volcano plot showing differentially expressed proteins (DEPs) between VD-CAG-treated (n=5) and untreated (n=5) bone marrow aspirate samples. Red and blue dots indicate significantly upregulated and downregulated proteins, respectively. **(B)** Venn diagram showing the overlap between differentially expressed proteins (DEPs, green) and differentially expressed genes (DEGs, red) identified in VD-CAG-treated versus untreated samples. A total of 97 molecules were found to be significantly dysregulated at both the transcriptomic and proteomic levels, highlighting the limited but biologically relevant concordance between mRNA and protein expression changes. **(C)** Pie chart illustrating the subcellular distribution of DEPs, with nucleus, cytoplasm, and mitochondrion proteins comprising the largest fractions. **(D)** InterPro (IPR) domain enrichment analysis of DEPs, highlighting the top enriched protein domains. **(E)** Gene Ontology (GO) term enrichment analysis of DEPs, showing significant enrichment in intracellular signaling, protein transport, and metabolic processes. **(F)** KEGG pathway enrichment analysis of DEPs, demonstrating significant involvement in metabolic and signaling pathways.

To further validate the biological relevance of the dysregulated proteins for VD-CAG response, we performed data-independent acquisition (DIA)-based quantitative proteomics on bone marrow aspirate samples from 5 paired patients (10 samples) (**Table 1**). Principal component analysis (PCA) of the proteomic data revealed a partial separation between the VD-CAG treated and untreated groups along the first principal component (PC1), which explained 35.72% of the variance (**Supplementary Figure 6**). While most samples from each group tended to cluster together, there was some overlap between groups and considerable within-group heterogeneity. This suggests that VD-CAG therapy induces global proteomic changes, but the response is variable among individuals, reflecting the biological heterogeneity of AML and patient-specific responses to treatment. In total, 6830 proteins were identified across all samples, among which 5783 proteins were quantified and compared (**Supplementary Table 3**). Differential expression analysis, using a threshold of ≥2.0-fold change (upregulation >2.0-fold or downregulation <0.5-fold) and *p* < 0.05, identified 447 significantly differentially expressed proteins (DEPs) in the Treated vs. Untreated comparison group (**Figure 5A**, **Supplementary Table 4**), including 159 significantly upregulated proteins and 288 significantly downregulated proteins. To further explore the relationship between transcriptomic and proteomic alterations following VD-CAG induction, we performed an integrative analysis of DEGs and DEPs. A Venn diagram illustrates the overlap between DEGs and DEPs, revealing that 97 genes were consistently dysregulated at both the mRNA and protein levels. In contrast, 350 DEPs were not accompanied by significant changes at the transcript level, while 3,810 DEGs did not show corresponding protein-level alterations. This limited overlap underscores the complexity of post-transcriptional and post-translational regulation in AML cells responding to VD-CAG therapy (**Figure 5B**).

Subcellular localization analysis showed that the majority of DEPs were distributed in the nucleus, cytoplasm, and mitochondria (**Figure 5C**). InterPro (IPR) domain enrichment analysis indicated that DEPs were significantly associated with domains involved in ribosomal function, DNA-dependent processes, and metabolic regulation (**Figure 5D**). GO enrichment analysis further highlighted terms related to intracellular signal transduction, protein transport, and metabolic processes (**Figure 5E**). KEGG pathway enrichment analysis revealed that DEPs were significantly involved in pathways such as glutamate metabolism, ribosome biogenesis, DNA replication, and several signaling pathways (**Figure 5F**).

### 3.8 RNA and protein expression profile of the 11-genes in prognostic signature

**Figure 6:**
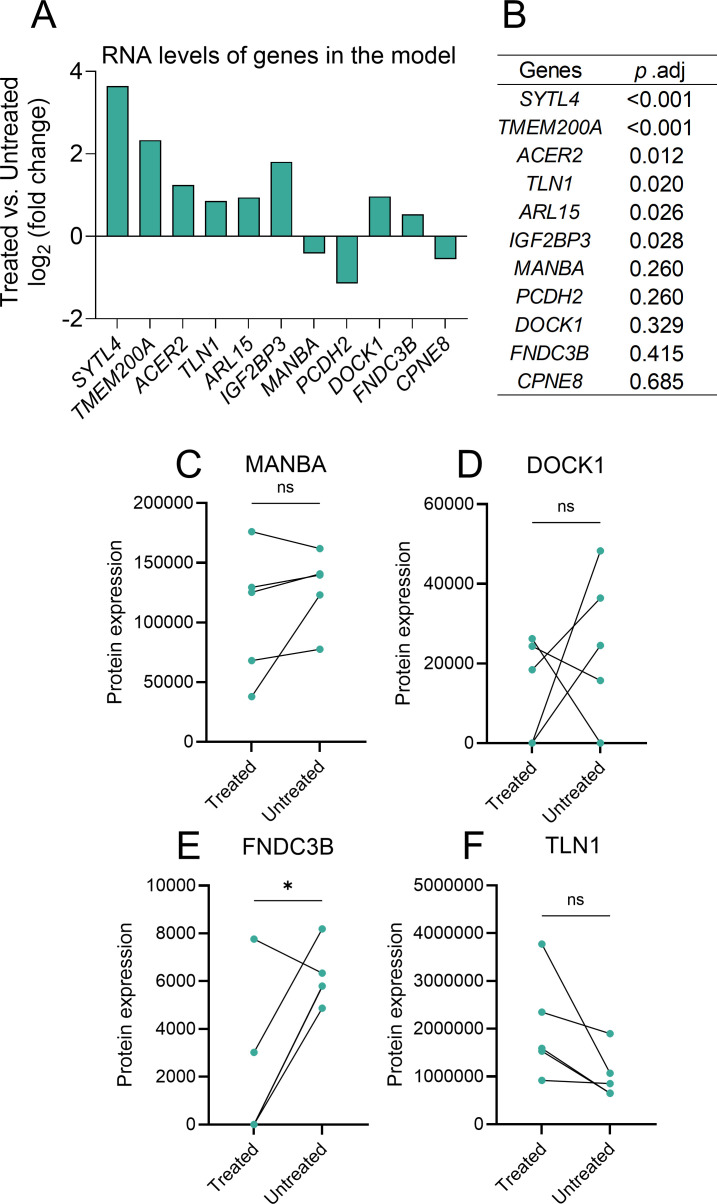
RNA and protein expression profile of the 11-genes in prognostic signature. **(A)** RNA expression changes (log2 fold change) of the 11-gene signature between treated and untreated groups. **(B)** Adjusted *p*-values for RNA expression differences of the 11 model genes between treated and untreated groups. **(C–F)** Protein expression levels of four model genes (MANBA, DOCK1, FNDC3B, TLN1) detected by LC-MS in treated and untreated groups.

RNA expression analysis of the 11-gene signature revealed that several genes, including *SYTL4, TMEM200A, ACER2, TLN1, ARL15*, and *IGF2BP3*, were significantly dysregulated at the transcript level after treatment (**Figures 6A, B**). However, only four proteins (MANBA, DOCK1, FNDC3B, TLN1) were detected in the proteomic dataset. Comparative analysis of protein expression between treated and untreated groups showed that *FNDC3B* was significantly downregulated following VD-CAG induction (**Figure 6E**, *p* < 0.05), while MANBA, DOCK1, and TLN1 did not reach statistical significance (**Figures 6C, D, F**).

## 4 Discussion

In this study, we conducted integrative transcriptomic and proteomic profiling of bone marrow aspirate samples from non-M3 AML patients undergoing VD-CAG induction therapy. Our findings demonstrate that VD-CAG induces profound transcriptomic reprogramming, characterized by the modulation of immune response, cell adhesion, and apoptotic pathways. Through exploratory WGCNA, we identified five candidate gene modules significantly correlated with CRc status, with the Chocolate4 module yielding an 11-gene prognostic signature that robustly stratified OS in both TCGA-LAML and Beat AML cohorts.

The superior performance of the Chocolate4-based signature highlights its potential as a reliable prognostic tool. This resilience may stem from the module’s enrichment in genes related to the malignant behaviors of AML. Specifically, TLN1 (talin-1, involved in integrin signaling) and DOCK1 (a guanine nucleotide exchange factor for Rac GTPases), which are critical for leukemia cell survival and chemoresistance (18–20). Knocking down of *TLN1* can inhibit the proliferation of AML cells and induce differentiation via the Talin1/P-AKT/CREB signaling pathway (18). Notably, DOCK1 has emerged as a key oncogenic driver promoting cell survival, growth, adhesion, and apoptosis resistance, particularly in subtypes with NPM1/cohesin mutations or MN1-driven phenotypes (19). By activating the Rac pathway and upregulating Notch signaling, DOCK1 facilitates leukaemogenesis, and its inhibition suppresses these pathways, ameliorating AML progression and positioning it as a promising therapeutic target (20). IGF2BP3, an RNA-binding protein in the signature, has been implicated in AML progression via regulating N6-methyladenosine (m(6)A) RNA methylation. It acts as a key m6A reader overexpressed in AML, promoting cell proliferation, survival, and disease progression by stabilizing critical mRNAs such as Sema4D (activating PI3K/AKT and ERK pathways to drive leukemogenesis) (21), DDX21 (cooperating with YBX1 to upregulate ULK1 and facilitate AML advancement) (22), and SENP1 (leading to HDAC2 de-SUMOylation and AKT signaling activation, exacerbating AML progression) (23).

Among the 11 genes included in the model, six (*SYTL4, TMEM200A, ACER2, TLN1, ARL15*, and *IGF2BP3*) were significantly upregulated in the treated group at the mRNA level. This transcriptomic upregulation was not fully consistent with the proteomic data, where only four model proteins (TLN1, MANBA, DOCK1, and FNDC3B) were detected in DIA proteomics and only FNDC3B was significantly downregulated in the treated samples compared to the untreated group. Notably, despite the lack of statistical significance for TLN1, its protein levels showed a consistent upward trend post-therapy. Such discrepancies likely reflect post-transcriptional regulation by VD-CAG therapy, such as ubiquitin-mediated proteolysis or miRNA interference (24, 25). Additionally, the lower sensitivity of DIA proteomics compared to RNA-seq may account for the non-detection of low-abundance signature proteins. Biologically, this discordance suggests that transcriptomic shifts capture rapid stress responses that may not immediately manifest as stable protein alterations during therapy-induced apoptosis. Clinically, this does not invalidate the prognostic model, as transcriptomic profiling is highly sensitive and technically standardized, making the 11-gene mRNA signature a practical tool for risk stratification.

Integrated pathway enrichment revealed both convergent and divergent molecular responses to VD-CAG. While RNA-seq highlighted shifts in immune modulation and apoptotic signaling, proteomic data emphasized metabolic reprogramming and ribosomal function. These discrepancies reflect differences in protein stability and turnover following therapy. Nevertheless, the overlap in immune and adhesion-related pathways suggests a coordinated response to VD-CAG. Based on these findings, we hypothesize a synergistic model for VD-CAG: G-CSF may prime leukemic blasts by mobilizing them from the bone marrow niche to sensitize them to cytarabine, aclarubicin, and decitabine. Concurrently, the modulation of MAPK and cytokine signaling may synergize with venetoclax to lower the apoptotic threshold.

The clinical applicability of the 11-gene signature could provide a dynamic layer of risk stratification beyond ELN 2022 guidelines (26, 27), specifically in: (1) Personalized Escalation: Patients identified as high-risk, even those within ‘favorable’ genetic categories, may benefit from early intensification. (2) Refined Transplant Allocation: The signature may clarify the necessity of allo-HSCT in first remission, particularly for intermediate-risk patients. (3) Enhanced Surveillance: Integrating this signature with MRD monitoring could identify patients at high transcriptomic risk for relapse.

Despite these promising results, several limitations remain. First, the small sample size warrants caution regarding WGCNA module stability. We mitigated this by rigorous external validation in Beat AML cohort. The fact that only the Chocolate4 module demonstrated consistent prognostic power suggests our framework effectively filtered out unstable artifacts. Second, the lack of direct functional validation remains a constraint; future studies are required to establish the mechanistic links between these genes and VD-CAG sensitivity. Third, the signature currently functions as a broad prognostic model rather than a VD-CAG-specific predictive biomarker, which requires further prospective verification.

## 5 Conclusion

This study elucidates the molecular landscape of VD-CAG response in non-M3 AML and establishes an 11-gene signature with strong prognostic utility. Future studies should validate this signature in prospective cohorts and explore its integration with targeted therapies to improve patient outcomes.

## Data Availability

The datasets presented in this study can be found in online repositories. The names of the repository/repositories and accession number(s) can be found below: https://ngdc.cncb.ac.cn/gsa-human/browse/HRA015465, HRA015465.

## Supplementary material

Supplementary Figure 1 Construction and validation of a prognostic gene signature for overall survival in non-M3 AML using Firebrick2 module genes. **(A)** Heatmap showing Firebrick2 module gene expression across non-M3 FAB subtypes in the TCGA-LAML cohort (n=133). **(B)** LASSO regression analysis in TCGA-LAML (non-M3) identifies a 7-gene prognostic signature from Firebrick2 module genes. Left: LASSO coefficient profiles; right: cross-validation plot for optimal lambda selection. **(C)** Risk score distribution, survival status, and expression heatmap of the 7 signature genes in TCGA-LAML (non-M3). **(D)** Kaplan-Meier survival curves for OS in TCGA-LAML (non-M3) stratified by the median 7-gene signature risk score. **(E)** Time-dependent ROC curves for the 7-gene signature in TCGA-LAML (non-M3), showing AUCs at 1, 3, and 5 years. **(F)** Kaplan-Meier survival curves for OS in the Beat AML validation cohort (non-M3) stratified by the median 7-gene signature risk score. **(G)** Time-dependent ROC curves for the 7-gene signature in the Beat AML validation cohort (non-M3), showing AUCs at 1 and 3 years.

Supplementary Figure 2 Construction and validation of a prognostic gene signature for overall survival in non-M3 AML using Pink3 module genes. **(A)** Heatmap showing Pink3 module gene expression across non-M3 FAB subtypes in the TCGA-LAML cohort (n=133). **(B)** LASSO regression analysis in TCGA-LAML (non-M3) identifies a 4-gene prognostic signature from Pink3 module genes. Left: LASSO coefficient profiles; right: cross-validation plot for optimal lambda selection. **(C)** Risk score distribution, survival status, and expression heatmap of the 4 signature genes in TCGA-LAML (non-M3). **(D)** Kaplan-Meier survival curves for OS in TCGA-LAML (non-M3) stratified by the median 4-gene signature risk score. **(E)** Time-dependent ROC curves for the 4-gene signature in TCGA-LAML (non-M3), showing AUCs at 1, 3, and 5 years. **(F)** Kaplan–Meier survival curves for OS in the Beat AML validation cohort (non-M3) stratified by the median 4-gene signature risk score. **(G)** Time-dependent ROC curves for the 4-gene signature in the Beat AML validation cohort (non-M3), showing AUCs at 1 and 3 years.

Supplementary Figure 3 Construction and validation of a prognostic gene signature for overall survival in non-M3 AML using Lightcyan module genes. **(A)** Heatmap showing Lightcyan module gene expression across non-M3 FAB subtypes in the TCGA-LAML cohort (n=133). **(B)** LASSO regression analysis in TCGA-LAML (non-M3) identifies a 13-gene prognostic signature from Lightcyan module genes. Left: LASSO coefficient profiles; right: cross-validation plot for optimal lambda selection. **(C)** Risk score distribution, survival status, and expression heatmap of the 13 signature genes in TCGA-LAML (non-M3). **(D)** Kaplan-Meier survival curves for OS in TCGA-LAML (non-M3) stratified by the median 13-gene signature risk score. **(E)** Time-dependent ROC curves for the 13-gene signature in TCGA-LAML (non-M3), showing AUCs at 1, 3, and 5 years. **(F)** Kaplan-Meier survival curves for OS in the Beat AML validation cohort (non-M3) stratified by the median 13-gene signature risk score. **(G)** Time-dependent ROC curves for the 13-gene signature in the Beat AML validation cohort (non-M3), showing AUCs at 1 and 3 years.

Supplementary Figure 4 Construction and validation of a prognostic gene signature for overall survival in non-M3 AML using Lavenderblush2 module genes. **(A)** Heatmap showing Lavenderblush2 module gene expression across non-M3 FAB subtypes in the TCGA-LAML cohort (n=133). **(B)** LASSO regression analysis in TCGA-LAML (non-M3) identifies a 2-gene prognostic signature from Lavenderblush2 module genes. Left: coefficient profiles for each gene; right: cross-validation curve for optimal lambda selection. **(C)** Distribution of risk scores, survival status, and expression heatmap of the 2 signature genes in TCGA-LAML (non-M3). **(D)** Kaplan-Meier survival analysis for overall survival in TCGA-LAML (non-M3) stratified by the median 2-gene signature risk score. **(E)** Time-dependent ROC curves for the 2-gene signature in TCGA-LAML (non-M3), with AUCs at 1, 3, and 5 years. **(F)** Kaplan-Meier survival analysis for overall survival in the Beat AML validation cohort (non-M3) stratified by the median 2-gene signature risk score. **(G)** Time-dependent ROC curves for the 2-gene signature in the Beat AML validation cohort (non-M3), with AUCs at 1 and 3 years.

Supplementary Figure 5 Incremental predictive value and clinical utility of the 11-gene signature beyond clinicogenetic factors. (A) Time-dependent C-index for the baseline Cox model (Age + Cytogenetics) and the expanded model (Age + Cytogenetics + 11-gene risk score) in the TCGA-LAML cohort; the ΔC-index and P value summarize the improvement in discrimination after adding the 11-gene risk score. (B) Net reclassification improvement (NRI) at the 3-year time horizon comparing the expanded model versus the baseline model in TCGA-LAML and Beat AML. Each dot represents an individual’s predicted 3-year event probability under the baseline (x-axis) and expanded (y-axis) models; colors indicate event status (event, non-event, censored). (C) Decision curve analysis (DCA) at 1-year and 3-year horizons in TCGA-LAML, showing net benefit across threshold probabilities for cytogenetic risk stratification, the 11-gene signature model, and default strategies (treat-all vs treat-none). (D) Time-dependent C-index for baseline versus expanded models in the Beat AML cohort with ΔC-index and P value. (E) Integrated discrimination improvement (IDI) at the 3-year horizon in TCGA-LAML and Beat AML. Curves show empirical cumulative distributions of the change in predicted 3-year event probability (Expanded − Baseline) for events (status = 1) and non-events (status = 0); IDI estimates with 95% CI and P values are shown. (F) Decision curve analysis (DCA) at 1-year and 3-year horizons in the Beat AML cohort.

Supplementary Figure 6 Principal component analysis (PCA) of the bone marrow proteomic profiles. The PCA score plot illustrates the global proteomic variation between VD-CAG treated (red circles, n=5) and untreated (blue circles, n=5) bone marrow aspirate samples. The horizontal and vertical axes represent the first two principal components (PC1 and PC2), explaining 35.72% and 12.87% of the total variance, respectively.

Supplementary Table 1 All dysregulated genes between the treated and untreated groups.

Supplementary Table 2 Details of five module genes for signature model construction.

Supplementary Table 3 All quantified proteins between the treated and untreated groups.

Supplementary Table 4 All dysregulated proteins between the treated and untreated groups.
