# Supplementary figures and images for: Discovery and validation of a robust 11-gene prognostic signature via integrative multi-omics profiling in VD-CAG-treated non-M3 AML
Source: Front Oncol. 2026 May 19;16:1801526. doi: 10.3389/fonc.2026.1801526 (PMC13225962; doi:10.3389/fonc.2026.1801526)

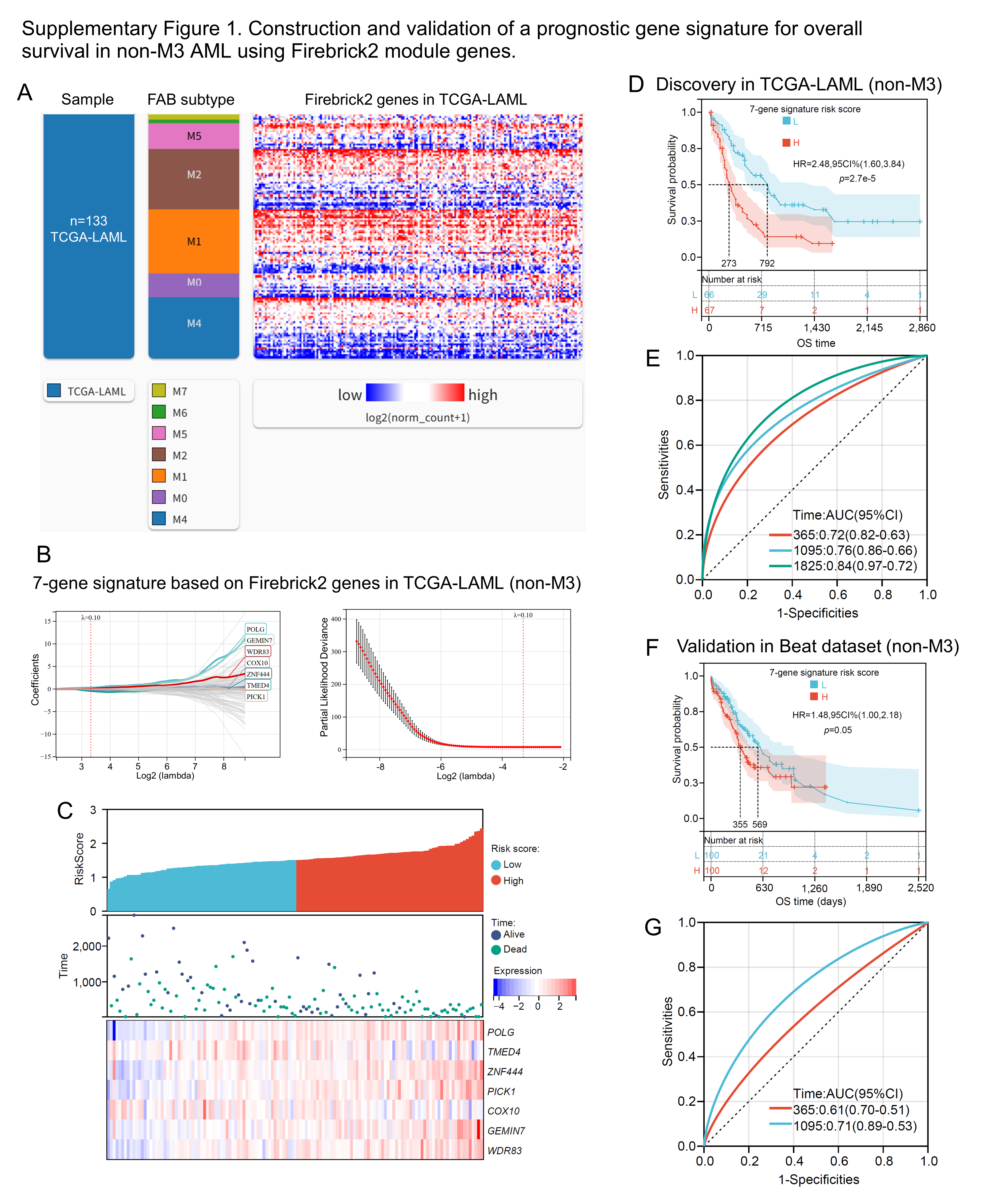

Supplement: Supplementary Figure 1 — Construction and validation of a prognostic gene signature for overall survival in non-M3 AML using Firebrick2 module genes. (A) Heatmap showing Firebrick2 module gene expression across non-M3 FAB subtypes in the TCGA-LAML cohort (n=133). (B) LASSO regression analysis in TCGA-LAML (non-M3) identifies a 7-gene prognostic signature from Firebrick2 module genes. Left: LASSO coefficient profiles; right: cross-validation plot for optimal lambda selection. (C) Risk score distribution, survival status, and expression heatmap of the 7 signature genes in TCGA-LAML (non-M3). (D) Kaplan-Meier survival curves for OS in TCGA-LAML (non-M3) stratified by the median 7-gene signature risk score. (E) Time-dependent ROC curves for the 7-gene signature in TCGA-LAML (non-M3), showing AUCs at 1, 3, and 5 years. (F) Kaplan-Meier survival curves for OS in the Beat AML validation cohort (non-M3) stratified by the median 7-gene signature risk score. (G) Time-dependent ROC curves for the 7-gene signature in the Beat AML validation cohort (non-M3), showing AUCs at 1 and 3 years. [file Image1.tif]

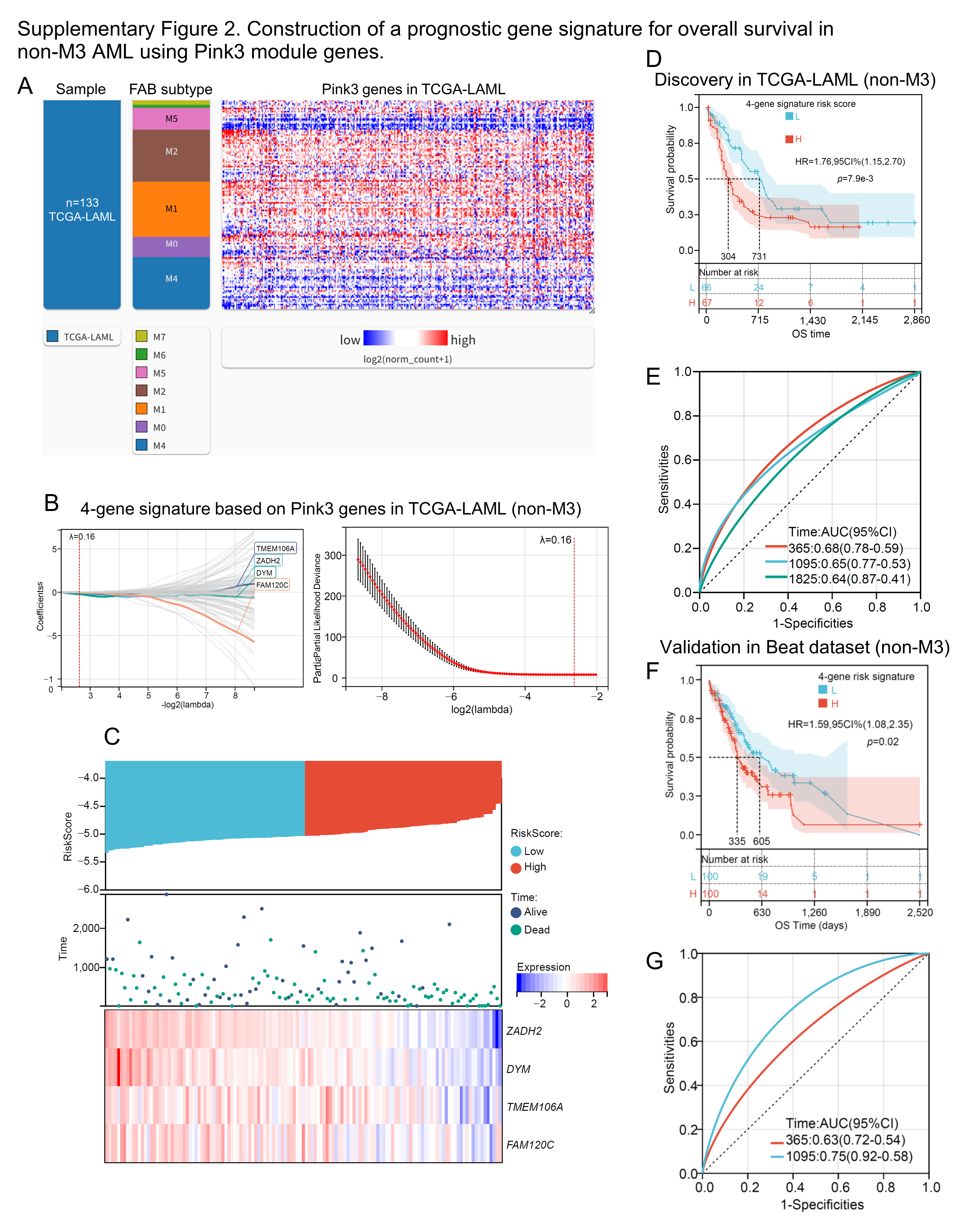

Supplement: Supplementary Figure 2 — Construction and validation of a prognostic gene signature for overall survival in non-M3 AML using Pink3 module genes. (A) Heatmap showing Pink3 module gene expression across non-M3 FAB subtypes in the TCGA-LAML cohort (n=133). (B) LASSO regression analysis in TCGA-LAML (non-M3) identifies a 4-gene prognostic signature from Pink3 module genes. Left: LASSO coefficient profiles; right: cross-validation plot for optimal lambda selection. (C) Risk score distribution, survival status, and expression heatmap of the 4 signature genes in TCGA-LAML (non-M3). (D) Kaplan-Meier survival curves for OS in TCGA-LAML (non-M3) stratified by the median 4-gene signature risk score. (E) Time-dependent ROC curves for the 4-gene signature in TCGA-LAML (non-M3), showing AUCs at 1, 3, and 5 years. (F) Kaplan–Meier survival curves for OS in the Beat AML validation cohort (non-M3) stratified by the median 4-gene signature risk score. (G) Time-dependent ROC curves for the 4-gene signature in the Beat AML validation cohort (non-M3), showing AUCs at 1 and 3 years. [file Image2.tif]

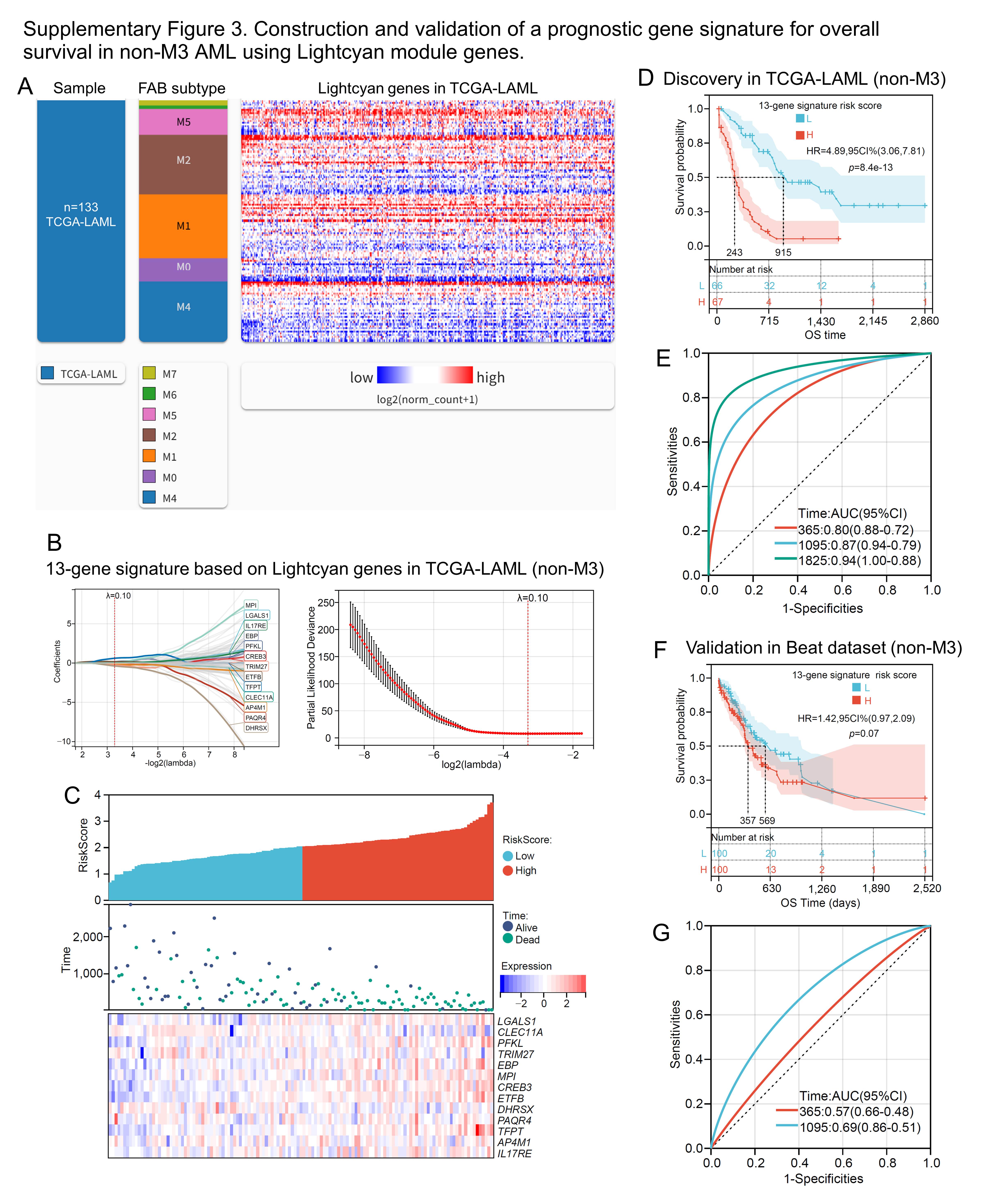

Supplement: Supplementary Figure 3 — Construction and validation of a prognostic gene signature for overall survival in non-M3 AML using Lightcyan module genes. (A) Heatmap showing Lightcyan module gene expression across non-M3 FAB subtypes in the TCGA-LAML cohort (n=133). (B) LASSO regression analysis in TCGA-LAML (non-M3) identifies a 13-gene prognostic signature from Lightcyan module genes. Left: LASSO coefficient profiles; right: cross-validation plot for optimal lambda selection. (C) Risk score distribution, survival status, and expression heatmap of the 13 signature genes in TCGA-LAML (non-M3). (D) Kaplan-Meier survival curves for OS in TCGA-LAML (non-M3) stratified by the median 13-gene signature risk score. (E) Time-dependent ROC curves for the 13-gene signature in TCGA-LAML (non-M3), showing AUCs at 1, 3, and 5 years. (F) Kaplan-Meier survival curves for OS in the Beat AML validation cohort (non-M3) stratified by the median 13-gene signature risk score. (G) Time-dependent ROC curves for the 13-gene signature in the Beat AML validation cohort (non-M3), showing AUCs at 1 and 3 years. [file Image3.tif]

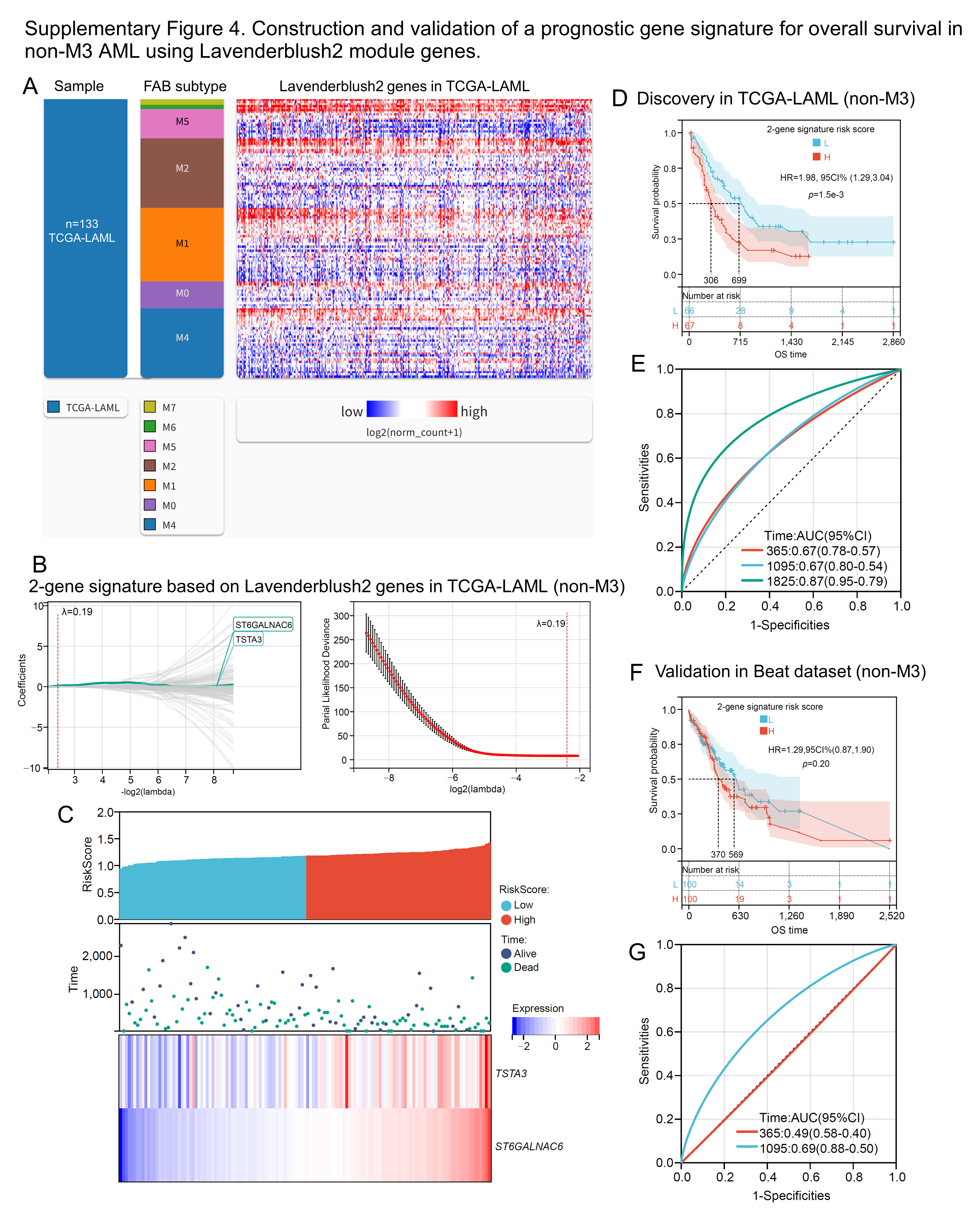

Supplement: Supplementary Figure 4 — Construction and validation of a prognostic gene signature for overall survival in non-M3 AML using Lavenderblush2 module genes. (A) Heatmap showing Lavenderblush2 module gene expression across non-M3 FAB subtypes in the TCGA-LAML cohort (n=133). (B) LASSO regression analysis in TCGA-LAML (non-M3) identifies a 2-gene prognostic signature from Lavenderblush2 module genes. Left: coefficient profiles for each gene; right: cross-validation curve for optimal lambda selection. (C) Distribution of risk scores, survival status, and expression heatmap of the 2 signature genes in TCGA-LAML (non-M3). (D) Kaplan-Meier survival analysis for overall survival in TCGA-LAML (non-M3) stratified by the median 2-gene signature risk score. (E) Time-dependent ROC curves for the 2-gene signature in TCGA-LAML (non-M3), with AUCs at 1, 3, and 5 years. (F) Kaplan-Meier survival analysis for overall survival in the Beat AML validation cohort (non-M3) stratified by the median 2-gene signature risk score. (G) Time-dependent ROC curves for the 2-gene signature in the Beat AML validation cohort (non-M3), with AUCs at 1 and 3 years. [file Image4.tif]

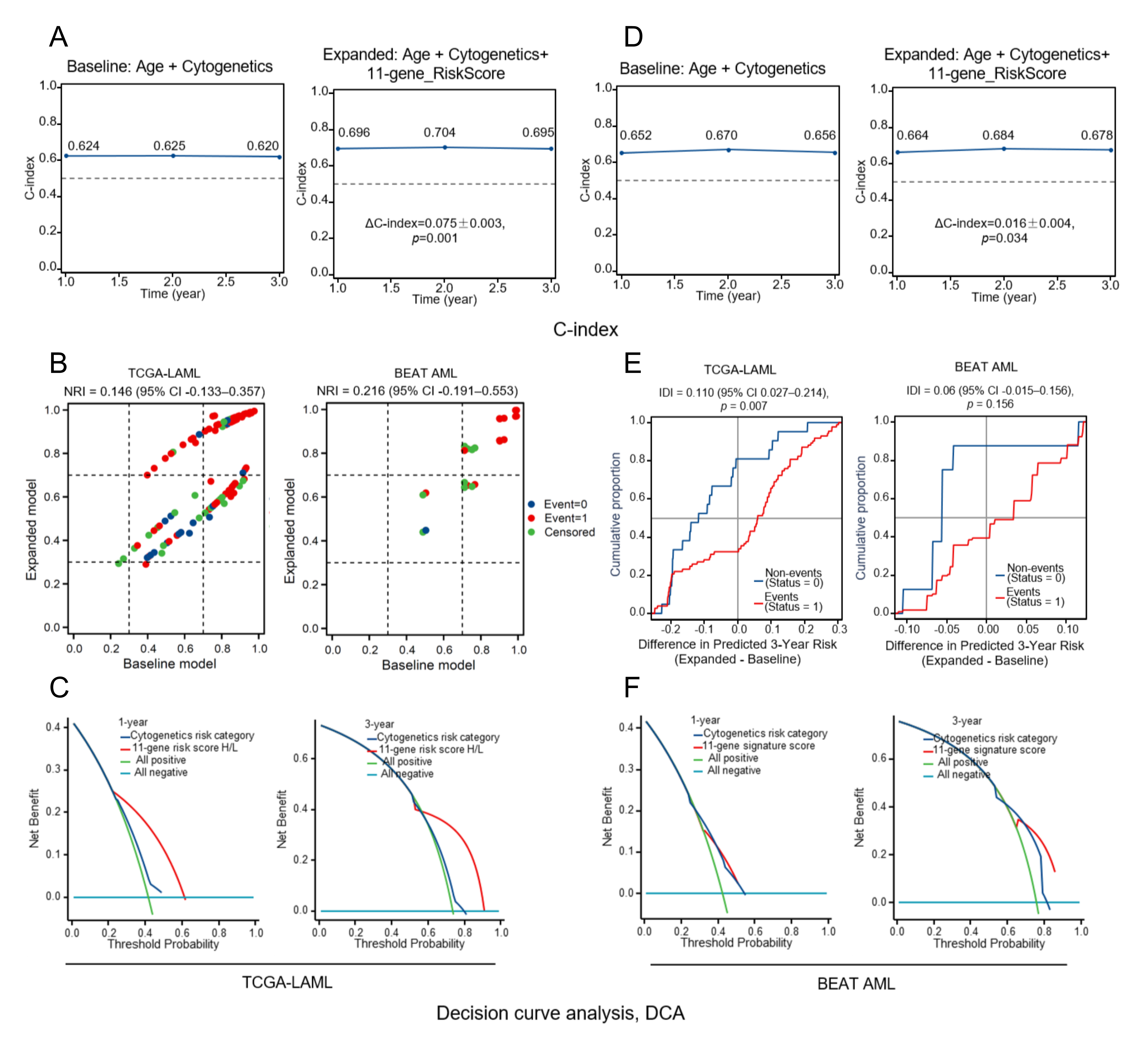

Supplement: Supplementary Figure 5 — Incremental predictive value and clinical utility of the 11-gene signature beyond clinicogenetic factors. (A) Time-dependent C-index for the baseline Cox model (Age + Cytogenetics) and the expanded model (Age + Cytogenetics + 11-gene risk score) in the TCGA-LAML cohort; the ΔC-index and P value summarize the improvement in discrimination after adding the 11-gene risk score. (B) Net reclassification improvement (NRI) at the 3-year time horizon comparing the expanded model versus the baseline model in TCGA-LAML and Beat AML. Each dot represents an individual’s predicted 3-year event probability under the baseline (x-axis) and expanded (y-axis) models; colors indicate event status (event, non-event, censored). (C) Decision curve analysis (DCA) at 1-year and 3-year horizons in TCGA-LAML, showing net benefit across threshold probabilities for cytogenetic risk stratification, the 11-gene signature model, and default strategies (treat-all vs treat-none). (D) Time-dependent C-index for baseline versus expanded models in the Beat AML cohort with ΔC-index and P value. (E) Integrated discrimination improvement (IDI) at the 3-year horizon in TCGA-LAML and Beat AML. Curves show empirical cumulative distributions of the change in predicted 3-year event probability (Expanded − Baseline) for events (status = 1) and non-events (status = 0); IDI estimates with 95% CI and P values are shown. (F) Decision curve analysis (DCA) at 1-year and 3-year horizons in the Beat AML cohort. [file Image5.tif]

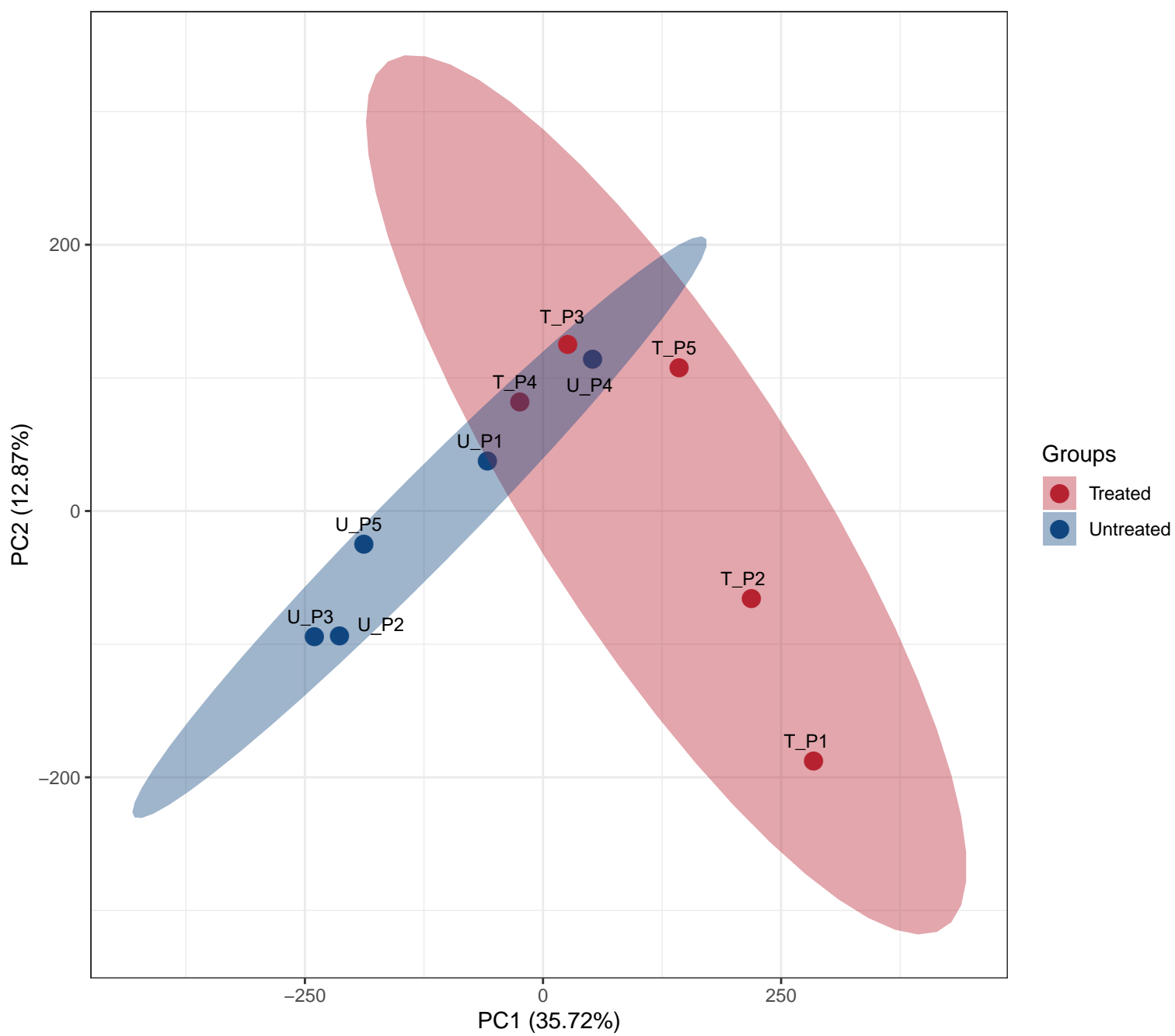

Supplement: Supplementary Figure 6 — Principal component analysis (PCA) of the bone marrow proteomic profiles. The PCA score plot illustrates the global proteomic variation between VD-CAG treated (red circles, n=5) and untreated (blue circles, n=5) bone marrow aspirate samples. The horizontal and vertical axes represent the first two principal components (PC1 and PC2), explaining 35.72% and 12.87% of the total variance, respectively. [file Image6.pdf]
